# A novel, multitargeted endogenous metabolic modulator composition impacts metabolism, inflammation, and fibrosis in nonalcoholic steatohepatitis-relevant primary human cell models

**DOI:** 10.1038/s41598-021-88913-1

**Published:** 2021-06-04

**Authors:** Nadine Daou, Andreu Viader, Murat Cokol, Arianna Nitzel, Manu V. Chakravarthy, Raffi Afeyan, Tony Tramontin, Svetlana Marukian, Michael J. Hamill

**Affiliations:** 1 Axcella Health Inc., 840 Memorial Drive, Cambridge, MA 02139 USA; 2 Jnana Therapeutics, Boston, MA USA; 3 Flagship Pioneering, Cambridge, MA USA; 4 Holmusk, New York, USA; 5 Valo Health, Cambridge, MA USA

**Keywords:** Non-alcoholic fatty liver disease, Non-alcoholic steatohepatitis, Biotechnology, Cell biology, Liver diseases, Hepatology, Liver, Hepatic stellate cells

## Abstract

Nonalcoholic steatohepatitis (NASH) is a complex metabolic disease of heterogeneous and multifactorial pathogenesis that may benefit from coordinated multitargeted interventions. Endogenous metabolic modulators (EMMs) encompass a broad set of molecular families, including amino acids and related metabolites and precursors. EMMs often serve as master regulators and signaling agents for metabolic pathways throughout the body and hold the potential to impact a complex metabolic disease like NASH by targeting a multitude of pathologically relevant biologies. Here, we describe a study of a novel EMM composition comprising five amino acids and an amino acid derivative (Leucine, Isoleucine, Valine, Arginine, Glutamine, and *N*-acetylcysteine [LIVRQNac]) and its systematic evaluation across multiple NASH-relevant primary human cell model systems, including hepatocytes, macrophages, and stellate cells. In these model systems, LIVRQNac consistently and simultaneously impacted biology associated with all three core pathophysiological features of NASH—metabolic, inflammatory, and fibrotic. Importantly, it was observed that while the individual constituent amino acids in LIVRQNac can impact specific NASH-related phenotypes in select cell systems, the complete combination was necessary to impact the range of disease-associated drivers examined. These findings highlight the potential of specific and potent multitargeted amino acid combinations for the treatment of NASH.

## Introduction

Nonalcoholic fatty liver disease (NAFLD) and its more severe fibroinflammatory presentation, nonalcoholic steatohepatitis (NASH), is an emerging public health threat paralleling the worldwide surge in obesity and related metabolic complications^1^. Currently, NAFLD is the most common chronic liver disease, and one-third of patients with NAFLD are estimated to progress to NASH^2^. Characterized by metabolic dysregulation, inflammation, and fibrosis^3^, NASH has a high risk of liver-related morbidity and mortality globally and is a leading cause of liver transplantation^4^. Despite the growing health and economic burden, there are no approved therapeutic options for NASH^4^. Therapeutic strategies currently being pursued reflect the heterogeneous pathogenesis of NASH and focus on specific biochemical pathways and nodes, including those associated with insulin resistance, lipotoxicity, endoplasmic reticulum oxidative stress, or mitochondrial and metabolic dysfunctions^3, 5^. Given the multifactorial nature of NASH^3, 5^, it is posited that approaches capable of safely and simultaneously modulating multiple pathways and drivers of this disease could yield sustainable disease-modifying results.

Endogenous metabolic modulators (EMMs) encompass a broad set of molecular families, including amino acids, related metabolites, and precursors. EMMs often serve as master regulators and signaling agents for metabolic pathways throughout the body and, as such, can impact liver function and health. For instance, research shows that glutamine helps maintain the intestinal mucosal integrity and limits gut permeability, reducing systemic and liver inflammation, and improving hepatic function^6, 7^. Arginine has shown promising results in enhancing liver function by modulating nitric oxide signaling that impacts vascular health and hepatic circulation^8, 9^. Its effect is also observed on the urea cycle to drive fatty acid oxidation^10^ and contribute to ammonia detoxification^11^. Arginine supplementation in patients with diabetes and obese individuals without diabetes resulted in improved glucose homeostasis, lipid parameters, and reduced central adiposity^12–14^. Supplementation with leucine, isoleucine, and valine (collectively referred to as branched-chain amino acids [BCAAs]), has been associated with improved clinical outcomes and positive modulation of systemic metabolism in patients with chronic liver disease^15–19^. While discrepancies exist in nonclinical literature examining the role of BCAAs on liver health and metabolism^20^, the positive effects of BCAA supplementation in patients with chronic liver disease are supported by a number of studies in rodent NASH models where BCAAs have been found to alleviate hepatic steatosis, and limit liver injury and fibrogenesis^21, 22^. Evidence to date indicates potent antioxidant properties of *N*-acetylcysteine (a precursor of glutathione) capable of decreasing NASH-relevant fibroinflammatory cascades^23^, resulting in a positive impact on liver function tests in patients with NASH^24, 25^.

While individual EMMs may be capable of affecting specific disease-relevant pathways, we hypothesized that combining EMMs with distinct modes of action into a single composition could modulate multiple biological pathways relevant to NASH. Indeed, we have previously shown that two higher-order combinations of EMMs (i.e., AXA1665 and AXA2678) could impact biology in complex diseases such as cirrhosis and muscle disuse atrophy^26, 27^. Based on our curation of previously published human and both published and unpublished nonclinical studies highlighting the ability of specific amino acids to impact distinct aspects of NASH pathology, we used our proprietary insights to design a novel EMM composition comprising five amino acids and an amino acid precursor (leucine [L], isoleucine [I], valine [V], arginine [R], and glutamine [Q], *N*-acetylcysteine [Nac]; LIVRQNac; referred to as AXA1125 in non-investigational new drug human studies). This combination has been found to demonstrate complementary modes of action across the three pathophysiological nodes of NASH: metabolic dysregulation, inflammation, and fibrosis. In two separate studies—a 12-week, multicenter, open-label clinical study^28, 29^, and a 16-week placebo-controlled, randomized, multi-arm clinical study^30^ dosing with AXA1125 improved key markers of metabolism, inflammation, and fibrosis, as well as relevant clinical measures in subjects with NAFLD with and without type 2 diabetes.

This study aimed to deconvolute the observed biological activity^28–30^ of LIVRQNac in a range of primary human cell model systems (hepatocytes, macrophages, and stellate cells in mono- and tri-culture) that replicate core NASH phenotypes. We report that LIVRQNac can simultaneously impact metabolic, inflammatory, and fibrotic processes similar to the clinical observations with AXA1125. Importantly, we show that while the individual constituent amino acids in LIVRQNac can impact specific NASH-relevant phenotypes in select cell systems, the full combination is necessary to achieve the desired impact on the range of disease drivers examined, highlighting the potential of specific and potent multitargeted amino acid combinations as novel NASH therapeutics.

## Results

### Adapting NASH-relevant in vitro model systems to reflect the amino acid composition of human plasma and enable testing of EMMs

We combined distinct amino acids with complementary modes of action to form LIVRQNac, to simultaneously target multiple metabolic pathways involved in maintaining normal liver function and health. In order to assess the ability of LIVRQNac to directly modulate drivers of NASH pathogenesis, we initially leveraged three primary human cell model systems that capture several NASH-relevant phenotypes: metabolic dysregulation in a primary human hepatocyte (PHH) model of lipotoxicity^31^, inflammation in a lipopolysaccharide (LPS)/interleukin-4 (IL-4)-stimulated primary human macrophage (PHM) model^32^, and fibrosis in a transforming growth factor-beta 1 (TGF-β1)-stimulated primary human hepatic stellate cell (HSC) model^33^.

The traditional culture media for PHHs (William's E medium; WEM [Gibco]), PHMs (Dulbecco's Modified Eagle Medium supplemented, DMEM [Gibco]), and HSCs (DMEM [Gibco]), however, contain amino acids at levels that do not reflect those in human plasma. This difference in the amino acid levels could likely alter the endogenous amino acid-related metabolism in these primary cells^34^ while also complicating the assessment of physiologically relevant effects associated with amino acid supplementation. Thus, we developed a set of custom WEM and DMEM culture media containing amino acids at concentrations that match those reported for plasma from healthy adults (values published in the Human Metabolome Database [HMDB]^35^; see Supplementary Table S1 and Methods for full details). The PHHs, PHMs, and HSCs cultured in their corresponding plasma amino acid-matched custom HMDB media retained a robust ability to respond to the stimuli used in our NASH in vitro models while also maintaining good overall cell health and viability as assessed by nuclear counts (Supplementary Fig. S1a–c).

Having established modified culture conditions for PHHs, PHMs, and HSCs that better reflect physiological amino acid levels, we then examined the effects of LIVRQNac supplementation on metabolic, inflammatory, and fibrotic phenotypes in NASH-relevant model systems utilizing each of these primary human cell types. In all experiments described below, we added the constituents of LIVRQNac at specified fold concentrations above normal plasma levels (10×–30× for LIVRQ; Nac is not endogenous in plasma and was added at 2.5–7.5 mM; see Supplementary Table S1). This approach mimics parenchymal and non-parenchymal liver cells' exposure to supraphysiological levels of amino acids that have been reported following their acute bolus supplementation in humans^36–38^.

### LIVRQNac improves metabolism in a PHH model of lipotoxicity

**Figure 1:**
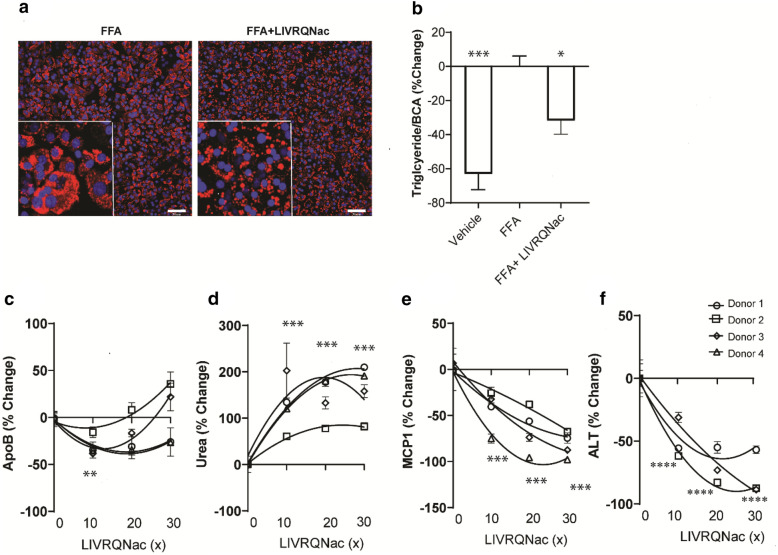
LIVRQNac improves dysregulated metabolism in a NASH-relevant primary human hepatocyte model of lipotoxicity. (**a**) Representative images of PHHs exposed to lipotoxic insult (FFA group) for 72 h in the presence (right) or absence (left) of LIVRQNac (30x relative to human plasma concentration for LIVRQ and 7.5 mM Nac; applied 24 h before lipotoxic insult) and stained with high-content screening LipidTOX red neutral lipid stain (red) to reveal lipid droplets and with Hoechst (blue) to show nuclei. Scale bars 74 µm. Insets have been magnified to provide greater clarity of the lipid phenotype. (**b**) Intracellular triglyceride levels measured in PHHs treated with vehicle (without lipotoxic insult) or exposed to lipotoxic insult (FFA group) and treated with LIVRQNac (30x relative to human plasma concentration for LIVRQ and 7.5 mM Nac, applied 24 h before lipotoxic insult) for 72 h post insult. Data are normalized to protein concentration assessed by BCA assay and shown as a mean percentage change relative to the FFA group. Data represent at least two technical replicates from at least three independent donors. Error bars represent ± SEM. *p < 0.05 versus FFA group, ***p < 0.001 versus FFA group. (**c**) ApoB, (**d**) urea, (**e**) MCP-1, (**f**) ALT levels measured in PHH supernatant exposed to lipotoxic insult (FFA group) and treated with either phosphate-buffered saline (0x) or LIVRQNac (10x–30x relative to human plasma concentration for LIVRQ and 2.5–7.5 mM Nac; applied 24 h before lipotoxic insult) for 72 h (ApoB and Urea, ALT) or 24 h (MCP-1) post insult. 0x corresponds to FFA-stimulated PHHs without additional LIVRQNac. Data are displayed as percent change relative to vehicle-treated PHHs exposed to lipotoxic insult (0x) and represent the mean of three technical replicates from four individual donors. Error bars represent ± SEM. **p < 0.01, ***p < 0.001, **** *p* < 0.0001 versus FFA (0x) group for all four donors combined. Analysis was performed using GraphPad Prism version 9.0.1 for Windows, GraphPad Software, San Diego, California USA, www.graphpad.com. The graphs were assembled using Adobe Illustrator CC 2019, www.adobe.com. *ApoB* apolipoprotein B, *ALT* alanine aminotransferase, *BCA* bicinchoninic acid, *FFA* lipotoxic insult (0.25 mM saturated free fatty acid [2:1 oleate: palmitate] + 1 ng/mL TNF-α), *MCP-1* monocyte chemoattractant protein 1, *Nac* *N*-acetylcysteine, *PHH* primary human hepatocyte, *SEM* standard error of mean, *TNF-α* tumor necrosis factor-alpha, *x* fold concentration.

Protracted lipid overload in the liver and ensuing lipotoxicity in the context of obesity and insulin resistance characterize the initial pathogenesis of NAFLD/NASH^39^. Thus, we initially examined the ability of LIVRQNac to modulate dysregulated lipid metabolism in a PHH model of lipotoxicity. As shown in Fig. 1a, PHHs exposed to lipotoxic insult (FFA: 0.25 mM; saturated free fatty acids [sFFA, 2:1 oleate: palmitate] + 1 ng/mL tumor necrosis factor-alpha [TNF-α]) for 72 h resulted in a robust accumulation of intracellular lipids as visualized by a neutral lipid stain. Treatment of these cells with LIVRQNac (30× for LIVRQ and 7.5 mM Nac) resulted in a visible shift in insult-induced intracellular lipid accumulation patterns from macrovesicular steatosis to microvesicular lipid droplets (Fig. 1a). Similarly, the enzymatic colorimetric assay for triglycerides showed that lipid challenge of PHHs for 72 h resulted in a significant elevation (62%, *p* < 0.001) in intracellular triglyceride levels compared to unstimulated PHHs (vehicle). Treatment of these cells with LIVRQNac (FFA + LIVRQNac), in turn, reduced intracellular triglyceride levels by 31% (*p* < 0.05) compared to the cells not treated with LIVRQNac (Fig. 1b).

Consistent with these reductions in insult-induced intracellular triglyceride accumulation, PHHs treated at a range of LIVRQNac concentrations (10×–30× for LIVRQ; 2.5–7.5 mM Nac) tended to secrete fewer very-low-density lipoproteins particles as determined by reduced supernatant apolipoprotein B100 (ApoB) levels (up to 34%; *p* < 0.01; Fig. 1c). Secretion of ApoB-containing very-low-density lipoprotein particles is a crucial mechanism by which liver hepatocytes regulate triglyceride levels to avoid steatosis^40, 41^ and, indeed, very-low-density lipoprotein secretion is dominantly stimulated by the availability of hepatic triglyceride^42, 43^. Modest variability in apolipoprotein supernatant levels was observed between donors, particularly at higher concentrations of LIVRQNac (Fig. 1c).

Increased flux through the urea cycle has been shown to drive hepatic fat oxidation^10^ and may also enhance ammonia detoxification, often compromised following chronic liver disease^11^. Thus, we next examined the impact of LIVRQNac on urea production by PHHs exposed to the lipotoxic insult. Treatment of lipotoxically-insulted PHHs with LIVRQNac at a range of concentrations (10×–30× for LIVRQ; 2.5–7.5 mM Nac) resulted in a concentration-dependent increase in PHH supernatant urea levels (up to 209%; *p* < 0.001) consistently across four different donors (Fig. 1d).

The impact of LIVRQNac on inflammatory responses of PHHs was examined by measuring monocyte chemoattractant protein-1 (MCP-1), a chemokine responsible for regulating the migration and infiltration of monocytes/macrophages^44^. Stimulation of PHHs with lipotoxic-insult for 24 h resulted in a significant elevation in MCP-1 supernatant levels (36%, *p* < 0.001; Supplementary Fig. S1). In turn, treatment with LIVRQNac at a range of concentrations (10×–30× for LIVRQ; 2.5–7.5 mM Nac) reduced MCP-1 levels in PHH supernatants in a concentration-dependent manner (up to 97.9%; *p* < 0.001; Fig. 1e) across all tested donors.

Furthermore, the impact of LIVRQNac on hepatocyte injury was examined by measuring the level of alanine aminotransferase (ALT), a biomarker correlating with inflammation and NAFLD activity score. Stimulation of PHHs with lipotoxic insult (FFA) slightly but significantly increased ALT level in the supernatant (17%, *p* < 0.05; Supplementary Fig. S1a). In turn, treatment with LIVRQNac at a range of concentrations (10×–30× for LIVRQ; 2.5–7.5 mM Nac) reduced ALT levels in PHH supernatants in a dose-dependent manner (up to 87%; *p* < 0.0001; Fig. 1f).

In summary, the results described above demonstrate the ability of LIVRQNac to modulate dysregulated lipid metabolism, urea cycle, and inflammation in a PHH model system that recapitulates many of the salient metabolic features of NASH.

### LIVRQNac modulates inflammatory responses in a PHM model of inflammation

**Figure 2:**
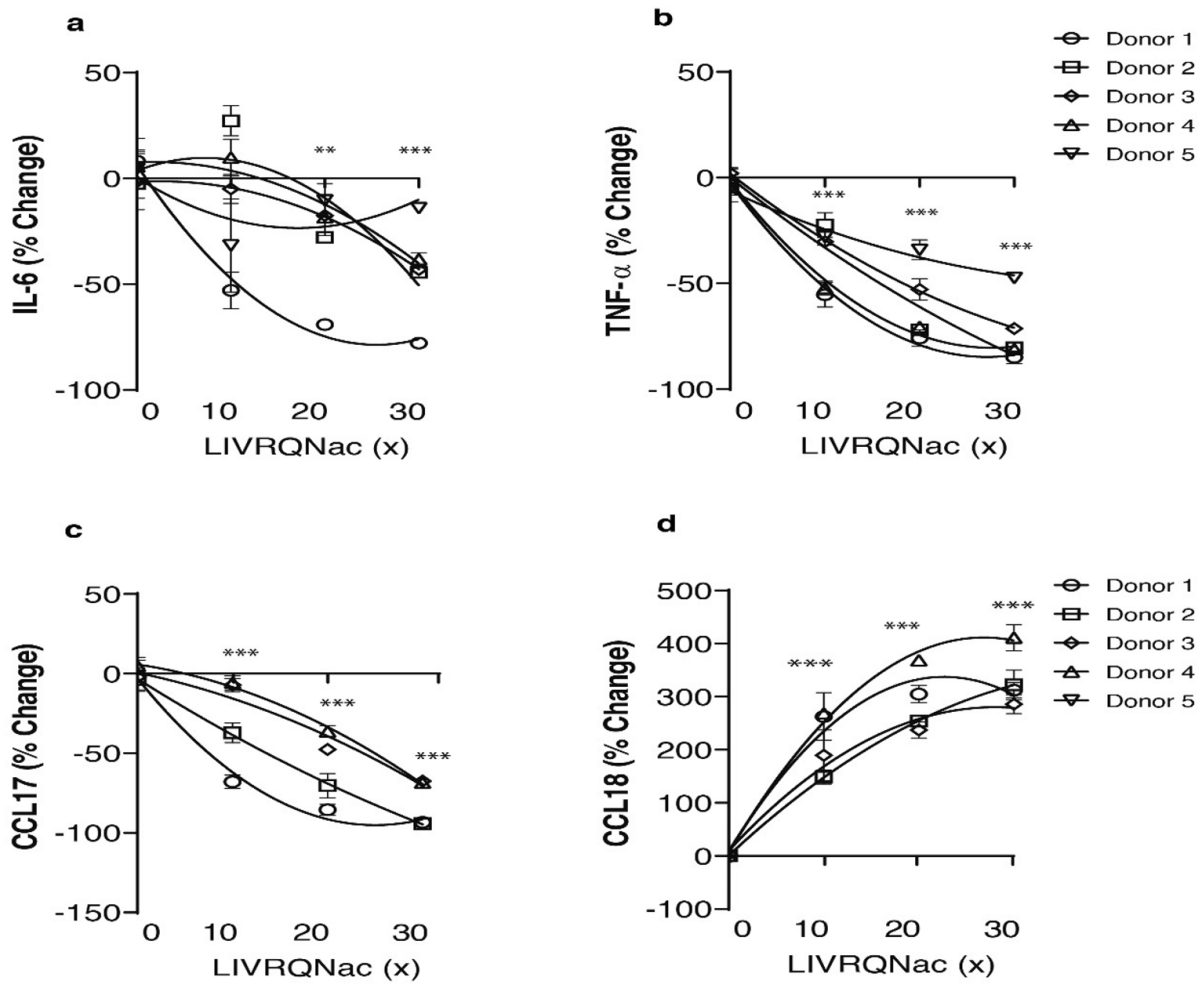
LIVRQNac reduces secreted levels of pro-inflammatory cytokines while inducing anti-inflammatory cytokine production in stimulated M1 and M2 primary human macrophages. (**a**) IL-6 and (**b**) TNF-α levels were measured in granulocyte–macrophage colony-stimulating factor derived M1 PHM supernatant following LPS (0.15 ng/mL) stimulation with or without the addition of LIVRQNac (10x–30x). Data are expressed in percentages relative to LPS-stimulated M1 (0x) and represent the mean of at least three technical replicates from five individual donors. **p < 0.01; ***p < 0.001 versus LPS (0x). (**c**) CCL17 and (**d**) CCL18 levels were measured in macrophage colony-stimulating factor-derived M2 PHM supernatants following IL-4 (1 ng/mL) stimulation with or without the addition of LIVRQNac (10x–30x). Data expressed in percentages relative to IL-4-stimulated M2 (0x) and represent the mean of at least three technical replicates from four individual donors. ***p < 0.001 versus IL-4 (0x). **Note:** 0x corresponds to LPS- or IL-4-stimulated M1 and M2 PHMs, respectively, treated with 1×HMDB media supplemented with an equivalent PBS vehicle volume. Error bars represent ± SEM. For (**a**–**d**): LIVRQNac was used at a range of concentrations (10x–30x of the human plasma concentration for LIVRQ and 2.5–7.5 mM Nac, respectively). Analysis was performed using GraphPad Prism version 9.0.1 for Windows, GraphPad Software, San Diego, California USA, www.graphpad.com. The graphs were assembled using Adobe Illustrator CC 2019, www.adobe.com. *CCL* C–C motif chemokine ligand, *HMDB* Human Metabolome Database, *IL* interleukin, *LPS* lipopolysaccharide, *Nac* *N*-acetylcysteine, *PBS* phosphate-buffered saline, *PHM* primary human macrophage, *SEM* standard error of the mean, *TNF-α* tumor necrosis factor-alpha, *x* fold concentration.

Liver inflammation, which is influenced by the balance between pro-inflammatory M1 and alternatively activated M2 macrophages, is a central node of NASH pathophysiology^45^. Having observed an impact of LIVRQNac on inflammatory chemokine production in PHHs, we leveraged in vitro models of M1- and M2-differentiated primary human macrophages to further explore the ability of this composition to impact inflammation. M1 (human leukocyte antigen-DR isotype; HLA-DR) and M2 (cluster of differentiation 163 and 206 [CD163 and CD206]) phenotype-specific markers measured by immunofluorescence staining (Supplementary Fig. S1b) confirmed that the cells had achieved M1 and M2 phenotype via sequential GM-CSF and LPS stimulation (0.15 ng/mL) and M-CSF and IL-4 (1 ng/mL) stimulation, respectively. In M1 PHMs, LPS stimulation for 24 h resulted in a significant increase in the levels of pro-inflammatory cytokines interleukin-6 (IL-6) and TNF-α (84.71% and 76.85%, respectively; *p* < 0.001; Supplementary Fig. S1) as measured in cell supernatants. Treatment with LIVRQNac (10×–30× for LIVRQ; 2.5–7.5 mM Nac) resulted in a concentration-dependent reduction in both extracellular IL-6 (up to 77.85%; *p* < 0.001) and TNF-α (up to 84.95%; *p* < 0.001) levels, consistently observed in M1 PHMs derived from five different donors (Fig. 2a,b).

In M2 PHMs, IL-4 stimulation induced the release of the pro-inflammatory chemokine CCL17 (89.96%; *p* < 0.001; Supplementary Fig. S1a) as well as the anti-inflammatory chemokine CCL18 (78.10%; *p* < 0.001; Supplementary Fig. S1a). Treatment of M2 PHMs with LIVRQNac (10×–30× for LIVRQ; 2.5–7.5 mM Nac) resulted in a concentration-dependent reduction in secreted CCL17 (up to 94.22%; *p* < 0.001; Fig. 2c) and a further concentration-dependent increase in secreted CCL18 (up to 411.32%; *p* < 0.001; Fig. 2d).

Taken together, these results demonstrate the ability of LIVRQNac to modulate M1 and M2 PHM responses in a NASH-relevant in vitro model of inflammation.

### LIVRQNac reduces fibrogenic markers to promote human HSCs quiescence in the fibrosis model

**Figure 3:**
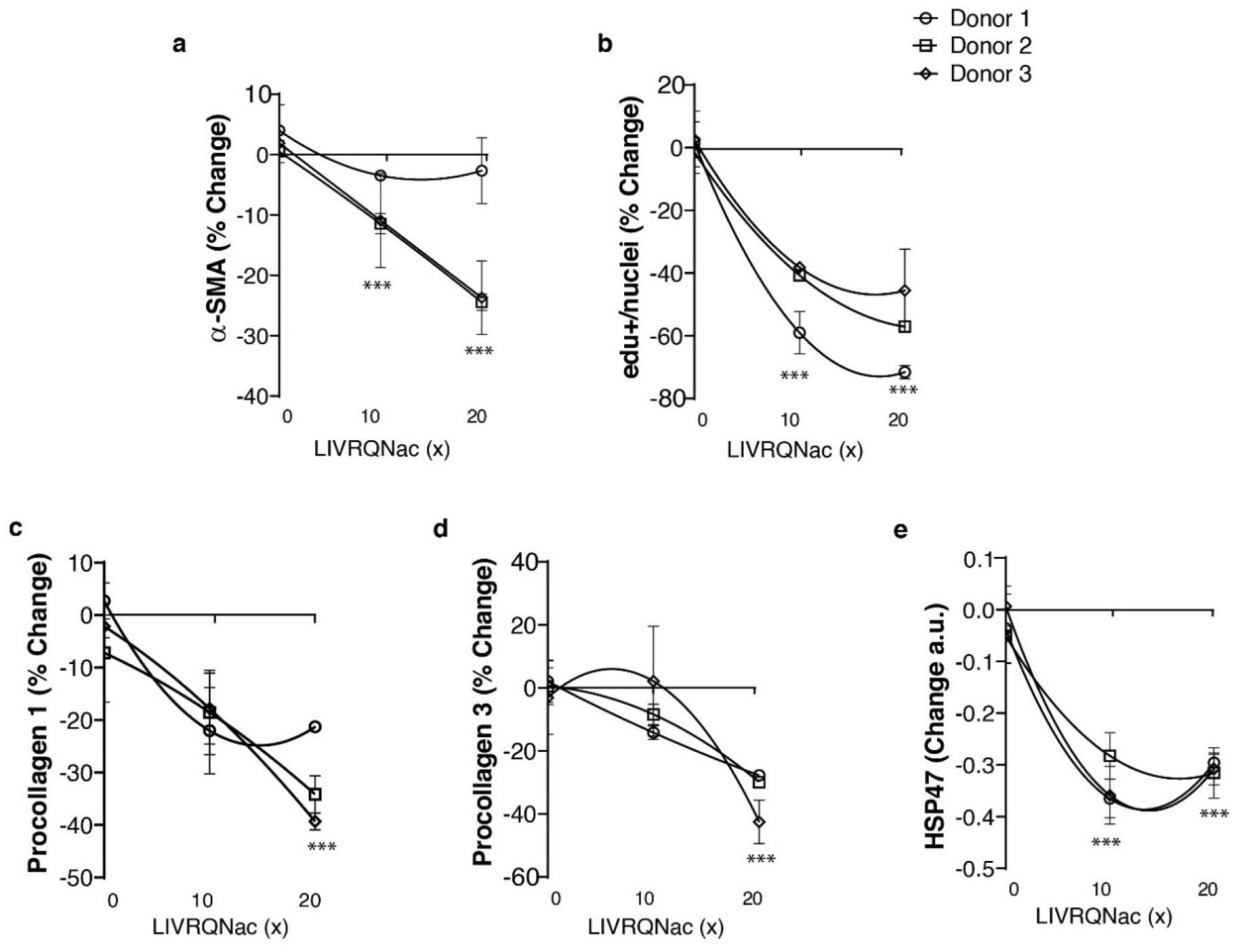
LIVRQNac reduces the induction of fibrogenic markers in TGF-β-treated human hepatic stellate cells. (**a**) α-SMA immunostaining and (**b**) labeled EdU positive intensity quantification from fixed and stained HSCs stimulated with TGF-β1 (3 ng/mL) and treated with either LIVRQNac (10x–20x) or with PBS vehicle (0x) for 24 h. Data is normalized to nuclei count and represented as a mean percentage change relative to TGF-β1-stimulated HSCs (0x) of at least three technical replicates from three independent donors. ***p < 0.001 versus TGF-β1 (0x) for all three donors combined. (**c**) Procollagen 1 and (**d**) procollagen 3 secreted levels measured in HSCs supernatant stimulated with TGF-β1 (3 ng/mL) and treated with either LIVRQNac or with PBS vehicle (0x) for 24 h. Data displayed as percent change relative to TGF-β1-stimulated HSCs (0x) represents the mean of at least three technical replicates from three independent donors. ***p < 0.001 versus TGF-β1 (0x) for all three donors combined. (**e**) HSP47 gene expression measured by qRT-PCR on RNA extracted from HSCs cells stimulated with TGF-β1 (3 ng/mL) and treated with either LIVRQNac (10x–20x) or with PBS vehicle (0x) for 24 h. GAPDH-normalized expression of HSP47 is represented as the mean of relative fold change to TGF-β1-stimulated HSCs (0x) of at least three technical replicates from three independent donors. ***p < 0.001 versus TGF-β1 (0x) for all three donors combined. **Note: **0x corresponds to TGF-β1-stimulated HSCs without additional LIVRQNac. Error bars represent ± SEM. LIVRQNac was used at a range of concentrations (10x–20x relative to human plasma concentration for LIVRQ and 2.5 mM and 5 mM Nac; applied 24 h before TGF-β1 stimulation). Analysis was performed using GraphPad Prism version 9.0.1 for Windows, GraphPad Software, San Diego, California USA, www.graphpad.com. The graphs were assembled using Adobe Illustrator CC 2019, www.adobe.com. *α-SMA* alpha-smooth muscle actin, *EdU* 5-ethynyl-2′-deoxyuridine, *GAPDH* glyceraldehyde 3-phosphate dehydrogenase, *HSC* primary human stellate cell, *HSP47* heat shock protein 47, *Nac* *N*-acetylcysteine, *PBS* phosphate-buffered saline, *qRT-PCR* quantitative reverse transcription-polymerase chain reaction, *RNA* ribonucleic acid, *SEM* standard error of mean, *TGF-β1* transforming growth factor beta-1, *x* fold concentration.

Liver fibrosis is the strongest predictor of NASH-related mortality^46^. Activation of HSCs, resulting in excessive deposition of extracellular matrix proteins and tissue scarring, is a well-established driver of fibrosis^47^. Thus, we next assessed the ability of LIVRQNac to impact fibrosis in HSCs stimulated with TGF-β1, a potent driver of the myofibroblastic transformation of these cells^48^. Stimulation of HSCs with TGF-β1 for 24 h resulted in a significant elevation in a number of fibrogenic proliferation and activation markers, including alpha-smooth muscle actin (α-SMA; 8% increase; *p* < 0.001; Supplementary Fig. S1), 5-ethynyl-2′-deoxyuridine (EdU) positive/nuclei (18% increase; *p* < 0.05; Supplementary Fig. S1), procollagen 1 and 3 (7% and 47% increase respectively; *p* not significant and 0.01, respectively; Supplementary Fig. S1), and heat shock protein 47 (HSP47; 38.53%; *p* < 0.001; Supplementary Fig. S1). In turn, LIVRQNac treatment (10×–20× for LIVRQ; 2.5–5 mM Nac) reduced the TGF-β-driven induction of α-SMA in HSCs by 25% (*p* < 0.001; Fig. 3a) in a concentration-dependent manner while also suppressing HSC proliferation as measured by EdU positive/nuclei by ≥ 60% (*p* < 0.001; Fig. 3b), which are two hallmarks of profibrogenic activation^47^. Similarly, a reduction in the levels of procollagen 1 (39%; *p* < 0.001) and procollagen 3 (42%; *p* < 0.001) was observed (Fig. 3c,d). Gene expression of HSP47, a molecular chaperone involved in the stabilization of collagen molecules^49^, was also significantly down-regulated (*p* < 0.001) with LIVRQNac treatment (10×–20× for LIVRQ; 2.5–5 mM Nac) (Fig. 3e). Taken together, these results suggest that LIVRQNac can reduce fibrogenic markers to promote HSC quiescence limiting their transition to a myofibroblastic phenotype.

### LIVRQNac treatment reduces fibroinflammatory markers in a multicellular in vitro model of NASH

**Figure 4:**
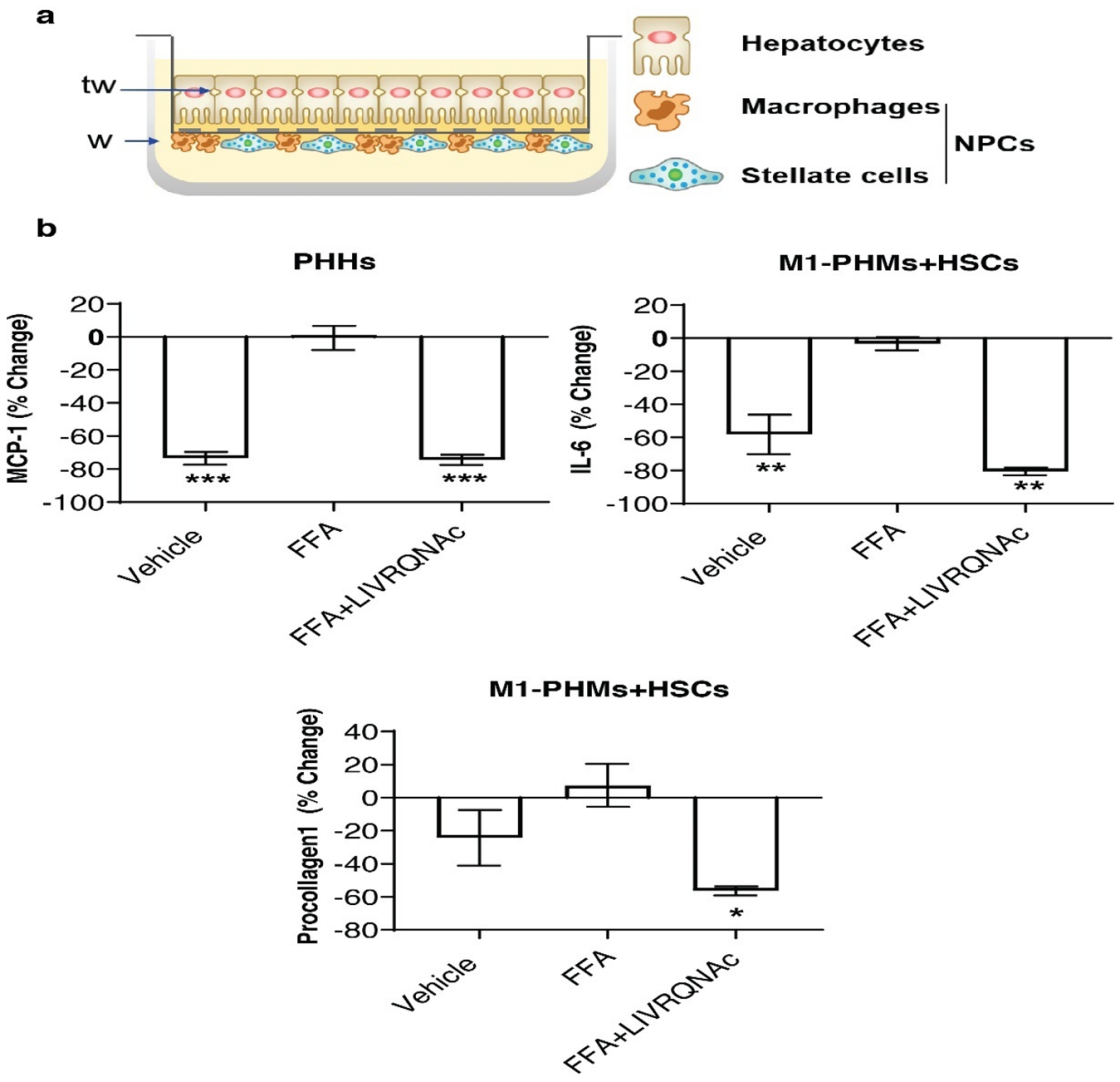
LIVRQNac treatment impacts markers of inflammation and fibrosis associated with NASH in a primary human multicellular system. (**a**) Cartoon scheme of liver multicellular in vitro system of primary human cells coculture in a transwell set up consisting of hepatocytes (top of the transwell: tw) and NPCs macrophages and HSCs (bottom of the tw; w). Adapted with permission from Ref. no. 31, American Society for Clinical Investigation using a CC BY license. (**b**) Secreted inflammatory markers (MCP-1, IL-6) and fibrosis marker (procollagen 1) were measured in the coculture supernatant collected from either the transwell (tw; PHHs) or the well (w; M1-PHMs + HSCs) side after 24 h treatment with either vehicle (without FFA) or with FFA, with or without additional LIVRQNac (30x of the human plasma concentration for LIVRQ and 7.5 mM Nac, applied 24 h before insult; FFA + LIVRQNac). Data expressed in percentage relative to FFA-treated cells represents the mean of two to three technical replicates from two individual donors. Error bars represent ± SEM. ***p < 0.001; **p < 0.01; *p < 0.05 versus FFA. Analysis was performed using GraphPad Prism version 9.0.1 for Windows, GraphPad Software, San Diego, California USA, www.graphpad.com. The graphs were assembled using Adobe Illustrator CC 2019, www.adobe.com. *FFA* lipotoxic insult (0.25 mM saturated free fatty acids [2:1 oleate: palmitate] + 1 ng/mL TNF-α), *IL* interleukin, *MCP-1* monocyte chemoattractant protein-1, *Nac* *N*-acetylcysteine, *NPC* nonparenchymal cell, *PHH* primary human hepatocyte, *SEM* standard error of the mean, *TNF-α* tumor necrosis factor-alpha, *tw* top of transwell, *w* bottom of transwell.

The complex pathophysiology of NASH reflects many interactions between parenchymal (i.e., hepatocytes) and non-parenchymal (e.g., Kupffer cells, HSCs) cells^50^. Given the robust effects of LIVRQNac on multiple pathophysiological features of NASH observed in several disease-relevant individual primary human cell systems, we further examined the impact of LIVRQNac on fibroinflammatory responses in a multicellular in vitro system that better mimics the cellular interactions of the in vivo microenvironments of the human liver. This multicellular in vitro system consisted of PHHs cocultured with M1 PHMs and HSCs separated by a synthetic porous membrane in transwell plates, allowing the three cell types to communicate through direct cell-to-cell contact and secreted factors (Fig. 4a).

In this multicellular model (Fig. 4a), lipotoxic stimulation for 24 h resulted in a significant elevation of IL-6 (58%; *p* < 0.01) and MCP-1 (73%; *p* < 0.001) inflammatory markers, as well as induction of the fibrosis marker procollagen 1 (24%; *p* = 0.3) (Fig. 4b). Importantly, 24 h treatment of the co-cultured cells with LIVRQNac (30× for LIVRQ and 7.5 mM Nac) significantly reduced the insult-driven induction of all measured fibroinflammatory markers (MCP-1 [80%; *p* < 0.001], IL-6 [74%; *p* < 0.01], and procollagen 1 [56%; *p* < 0.05]) (Fig. 4b). These effects were similar to those observed in single-cell systems (Figs. 1e, 2a, 3c). The data from the corresponding cell side of the well/transwell (i.e., MCP-1 as measured in supernatant from the PHH side; IL-6 and procollagen 1 as measured from the PHM and HSC side) are shown in Fig. 4. The effects of LIVRQNac on fibroinflammatory markers were equivalent in supernatant collected from either the well or transwell sides, confirming the free exchange of secreted factors in this system.

These findings demonstrate the ability of LIVRQNac to modulate inflammation and fibrosis in a multicellular physiologically relevant in vitro model of NASH, further bolstering the observations previously made in single-cell systems. Furthermore, these results provided additional support for the direct effect of LIVRQNac on fibro-inflammation, this time in the context of a lipotoxic insult versus TGF-β stimulation used in HSC monoculture and LPS treatment in M1 macrophages (Figs. 2, 3).

### Individual amino acid constituents of LIVRQNac contribute to the specific activities of this composition but are insufficient to elicit its full range of biological effects

Having confirmed the multifactorial activity of LIVRQNac and its ability to impact metabolism-, inflammation-, and fibrosis-related phenotypes across multiple NASH-relevant primary human single- and multi-cellular systems, we assessed the relative contribution of its constituent amino acids to the overall activity of the complete composition. To this end, we took a comprehensive approach and compared the effects of LIVRQNac on a number of the disease-relevant phenotypes described in our PHH, PHM, and HSC cell models to those elicited by its constituent amino acids (L, I, V, R, Q, Nac) or by the combination of its three BCAA components (LIV). To broadly compare the activity of LIVRQNac to that of its constituent amino acids across multiple phenotypes measured in different cell systems in a quantitative and unbiased way, we devised a desirability-based, multi-objective optimization, global ranking approach (META-rank) similar to a method previously utilized in the optimization of antibiotic combinations^51^.

**Figure 5:**
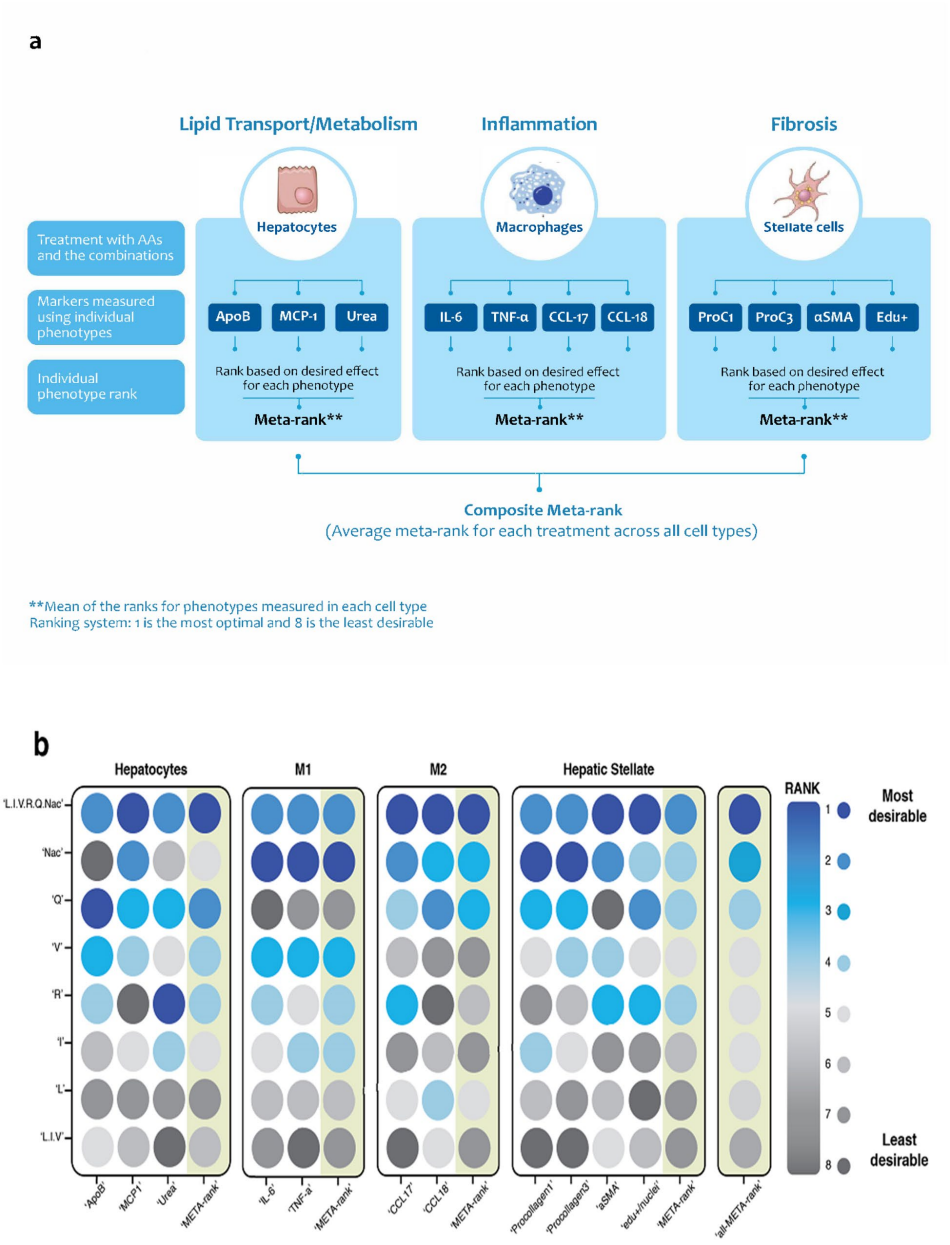

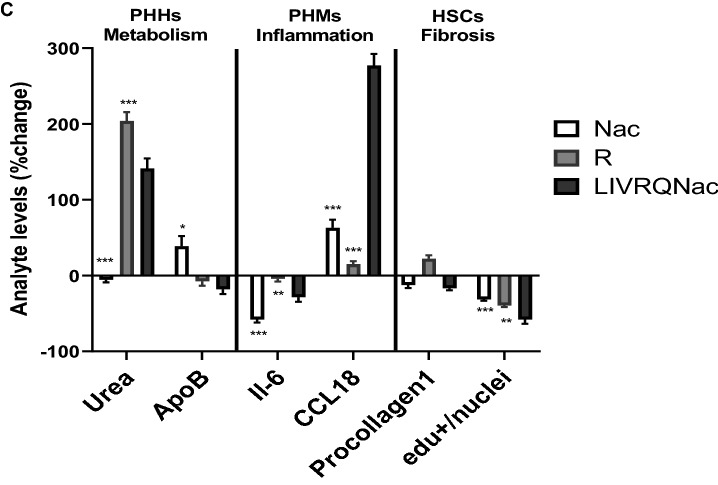
Treatment with LIVRQNac is necessary to achieve the desired impact on NASH-relevant metabolic, inflammatory, and fibrotic phenotypes across multiple primary human cell systems. (**a**) Schema to describe the global ranking approach to assess the effectiveness of LIVRQNac versus the individual amino acids of the composition. The figure was designed using Adobe Illustrator CC 2019, www.adobe.com. (**b**) Heatmap shows phenotype effects in four different cell types (Hepatocytes, M1 and M2 macrophages, and Hepatic stellate cells) after being treated with the adequate stimuli for each of the cell types (e.g., sFFA [0.25 mM] + TNF-α [1 ng/mL]) for hepatocytes; LPS (0.15 ng/mL) for M1; IL4 (1 ng/mL) for M2 and TGF-β (3 ng/mL) for Hepatic stellate cells) with or without additional LIVRQNac, L, I, V, R, Q, Nac, and LIV at 20x concentration relative to the normal plasma levels for LIVRQ, L, I, V, R and Q and 5 mM for Nac. For each phenotype, a rank was assigned to each treatment according to their desired effect, 1 being the best treatment for that phenotype, and 8 being the worst. For each cell type, a META-rank was calculated, which is the mean of the ranks for the phenotypes measured in the cell type. Treatments with low meta-ranks are most beneficial across all the considered phenotypes for the cell type. For all cell types, an all-META-rank was calculated, which is the mean meta-rank of all cell types. (**c**) Examples of Nac and R effects compared to LIVRQNac on selected NASH-related endpoints implicated in metabolism, inflammation, and fibrosis. Error bars represent ± SEM. ***p < 0.001; **p < 0.01; *p < 0.05. Analysis was performed using GraphPad Prism version 9.0.1 for Windows, GraphPad Software, San Diego, California USA, www.graphpad.com. The graphs were assembled using Adobe Illustrator CC 2019, www.adobe.com. *α-SMA* α-smooth muscle actin, *CCL* C–C motif chemokine ligand, *EdU* 5-ethynyl-2′-deoxyuridine, *I* isoleucine, *IL-4* interleukin-4, *IL-6* interleukin-6, *L* leucine, *LPS* lipopolysaccharide, *MCP-1* monocyte chemoattractant protein 1, *Nac* *N*-acetylcysteine, *Q* glutamine, *R* arginine, *sFFA* saturated free fatty acid, *TGF-β* tumor growth factor-beta, *TNF-α* tumor necrosis factor-alpha, *V* valine.

Figure 5a details the methodology used to understand the effects of individual amino acids of LIVRQNac versus the complete composition. For each phenotype within a given cell model system, we assigned a rank to each amino acid treatment according to the desirability of its elicited effect, with 1 being the most desirable treatment for each specific phenotype (e.g., Nac for IL-6) and 8 being the least desirable (e.g., Q for IL-6). For each cell type, we then calculated a META-rank (mean of the ranks) for the phenotypes measured in each cell type (e.g., IL-6 and TNF-α for M1 PHMs). Treatments with low META-ranks were the most beneficial across all the considered phenotypes for a given cell model. Finally, we calculated a composite all-META-rank (average META-rank for each treatment across all the cell types), which captured the optimal amino acid treatment tested across all phenotypes and all cellular systems. A heatmap of the ranks for each phenotype, META-ranks for each cell type, and all-META-rank across cell types are shown in Fig. 5b. The full dataset used to generate this META-rank is detailed in Supplementary Table S2.

Focusing on individual phenotypes measured in specific cell systems, we confirmed some of the expected biological activities of the individual amino acid constituents of LIVRQNac. For example, consistent with the biochemical role of arginine in the urea cycle and ammonia detoxification^11^, treating PHHs with this amino acid in isolation resulted in a robust increase in urea production (rank 1) while having minimal effects (rank 3–8) on other metabolic or inflammatory PHH phenotypes (i.e., ApoB and MCP-1 secretion) or fibroinflammatory phenotypes in PHM and HSC model systems (Fig. 5b,c, Supplementary Table S2). Similarly, in agreement with the well-established role of Nac in modulating fibroinflammatory cascades^23^, we observed that treatment with this amino acid precursor robustly reduced the levels of procollagen 1 and 3 (rank 1), proliferation in HSCs (rank 3), and pro-inflammatory cytokine IL-6 in M1 PHMs (rank 1), while elevating the anti-inflammatory chemokine CCL18 (rank 3) in M2 PHMs. These effects of Nac on fibroinflammation were seen in the absence of substantial metabolic effects in PHHs (rank 6 and 8 for urea and ApoB phenotypes in PHHs, respectively; Fig. 5b,c, Supplementary Table S2). These results showed that neither arginine nor Nac in isolation could impact the range of phenotypes to the same degree as measured across all three cell systems (Fig. 5b,c, Supplementary Table S2).

Interestingly, LIVRQNac retained its different amino acid constituents' activity on phenotypes examined across PHH, PHM, and HSC model systems. For example, like arginine, LIVRQNac robustly induced urea production in PHHs while maintaining Nac's ability to impact fibroinflammatory phenotypes in PHMs and HSCs (Fig. 5b,c, Supplementary Table S2). While the ability of LIVRQNac to modulate phenotypes of interest in these model systems was not identical to that of its individual constituents (e.g., the slightly attenuated effect on IL-6 secretion in M1 PHMs compared to Nac, the best-performing amino acid constituent for this phenotype), the results were generally either of similar magnitude or even more pronounced (e.g., robust induction of CCL18 in PHMs compared to Nac, the best-performing amino acid constituent for this phenotype) (Fig. 5b,c).

Overall, LIVRQNac scored low-rank numbers for specific phenotypes within each cell type while also scoring low META-rank numbers across the four cell model systems examined (Fig. 5b). The ability of LIVRQNac to significantly impact metabolic, inflammatory, and fibrotic parameters (Fig. 5c) resulted in this composition globally outperforming its individual amino acid constituents and achieving the best all-META-rank score (Fig. 5b).

Taken together, the data and META-rank analysis approach described above demonstrate that while individual constituent amino acids of LIVRQNac can impact specific NASH-relevant phenotypes limited to select cell systems, the complete combination is necessary to address the full range of dysfunction across all relevant cell types involved in the pathogenesis of NASH.

## Discussion

NASH is a systemic and multifactorial disease that benefits from therapeutic interventions that simultaneously target multiple drivers of its pathogenesis. EMMs that are inherent to the body and act as signaling agents for metabolic pathways may provide a safe way to impact NASH's multifactorial pathophysiology. We designed a novel EMM composition comprising five amino acids and an amino acid derivative (LIVRQNac) that has been found to coordinately support pathways related to metabolism, inflammation, and fibrosis. We have also demonstrated the ability of this composition (as AXA1125) to impact liver biology and function in both a multicenter, open-label clinical study^28, 29^, as well as in a placebo-controlled, randomized, multi-arm clinical study^30^.

The present work shows that LIVRQNac can produce consistent, reproducible, and potentiating effects across multiple NASH-relevant phenotypes modeled in primary human cell systems. Notably, we observed that while the individual constituent amino acids in LIVRQNac could impact specific NASH-relevant phenotypes in select cell systems, the complete combination was necessary to impact the range of disease drivers examined across the different cellular systems. This highlights the potential of specific and potent multitargeted amino acid combinations as a novel treatment option for NASH.

Amino acids and their metabolites and precursors are central to cellular physiology, often acting as metabolic master regulators and signaling agents. Not surprisingly, amino acids and related molecules have a rich history of evaluation for therapeutic purposes^52^, most commonly as single-agent interventions. For example, glutamine is currently approved to treat sickle cell disease and has been shown to reduce sickle cell-related pain and acute chest syndrome episodes^53, 54^. Similarly, Nac is used clinically to treat hepatotoxicity caused by acetaminophen overdose^55^. In a few cases, combinations of amino acids have been evaluated to treat specific diseases^56, 57^ by targeting single biology. For example, a stable salt combination of the urea cycle amino acids, l-Ornithine, and l-aspartate, has been used to address hyperammonemia^56^. Additionally, sarcopenia in muscle-related conditions has been targeted with combinations of essential amino acids^57^. The results from this study confirm the impact of individual amino acids (i.e., arginine, glutamine, and Nac) or a subset of combinations of these amino acids (BCAAs) on particular biologies and biochemical pathways. We observed that arginine and BCAAs could affect the urea cycle and lipid metabolism in PHHs, respectively (Fig. 5b,c, Supplementary Table S2). In turn, glutamine modulated CCL18 secretion in M2 PHMs, while Nac impacted fibroinflammatory cascades in PHM and HSC model systems (Fig. 5b,c, Supplementary Table S2). We also observed that individual components of BCAA (specifically L and I) were not as impactful as the other amino acid constituents for the set of endpoints measured in these in vitro models. However, L still demonstrated relevant activity on the CCL18, and I on Urea, TNF-α, and Procollagen 1; I also demonstrated activity on Procollagen 3 although not pronounced as compared to others (Supplementary Table S2). BCAAs are well known to have a robust metabolic activity. It is well established through published research that L, I, and V have effects on several NASH relevant pathways including protein synthesis, glucose homeostasis, anti-obesity, and nutrient-sensitive signaling pathways, e.g., phosphoinositide 3-kinase-protein kinase B (PI3K-AKT), and mammalian target of rapamycin (mTOR)^58, 59^. While discrepancies exist in nonclinical literature examining the role of BCAAs on liver health and metabolism^20^, there are several evidences reporting beneficial effects of BCAA supplementation on dysregulated metabolism and clinical outcomes in the context of chronic liver disease^15–19, 21, 22^ which supported their inclusion in AXA1125. Hence, we included them in our in vitro studies to compare the full combination of AXA1125 (LIVRQNac) to its single constituents and deconvolute the effect of this composition on NASH biology as represented by the relevant phenotypes reported in the META-rank analysis.

Our work recalibrates the traditional view of amino acids as single therapeutic agents best suited to address a single defined dysregulated biology. Given the diverse and complementary nature of the biological effects of different amino acids as well as their unique safety profile even when taken chronically at high doses, combined with our observations with a different amino acid combination in vivo and in vitro such as this study, we hypothesize that this EMM class is particularly amenable to higher-order combinations designed to tackle complex diseases of multifactorial pathogenesis. The results from our research exemplify how combining amino acids with robust metabolic activity (i.e., BCAAs and arginine; Fig. 5b, Supplementary Table S2) with others capable of impacting fibroinflammatory cascades (i.e., glutamine and Nac; Fig. 5b, Supplementary Table S2), resulted in a composition—LIVRQNac—capable of modulating the main drivers of NASH pathogenesis in a multitargeted manner. This observed multifactorial activity of LIVRQNac is consistent with the effects of two previously published higher-order combinations of amino acids, AXA1665, and AXA2678, on specific biologies implicated in cirrhosis and muscle disuse atrophy^26, 27^.

Interestingly, while deconvoluting the relative contribution of the individual amino acid constituents of LIVRQNac to the composition's overall activity (Fig. 5), we observed several unexpected positive interactions that potentiated the combination's overall effects. The impact of LIVRQNac on phenotypes such as the suppression of MCP-1 secretion by lipotoxically-insulted PHHs, the induction of CCL18 released by stimulated M2 PHMs, or the inhibition of TGF-β1-induced HSC proliferation was more significant than that observed for any of the individual amino acid constituents (Fig. 5c, Supplementary Table S2). Given that amino acid metabolic pathways are tightly interconnected, combinations of certain amino acids could display additive or even synergistic effects, highlighting a key strength of higher-order amino acid compositions to modulate dysregulated cellular pathways maximally. We anticipate that progress in network biology systems-level approaches, which provide a complete view of crosstalk among drivers of multifactorial diseases and of interacting EMM activities, together with improved combinatorial design strategies, will significantly expedite the rational design of EMM compositions to more comprehensively address complex diseases^52^.

Quantitative integration of the effects of multitargeted EMM compositions across multiple phenotypes and model systems poses a challenge when comparing and optimizing the activities of individual or of higher-order EMM combinations. Multi-objective optimization is, in fact, a common problem in drug design, where numerous pharmaceutically important objectives must be adequately satisfied in parallel, and the issues are particularly acute in the context of polypharmacological interventions^60^. Our META-rank approach offered a simple desirability-based, multi-objective optimization method to integrate the overall effect of EMM compositions across heterogenous bioactivity datasets. As shown above, META-rank enabled the direct comparison of the effects of LIVRQNac to its individual constituent amino acids in a global fashion across three disparate disease nodes: metabolic dysregulation, inflammation, and fibrosis. The resulting quantitative and unbiased metric supported the superior overall activity of LIVRQNac relative to that of its constituent amino acids or small combinations (Fig. 5b). META-rank is thus an example of the type of multi-objective optimization tool that, when combined with in silico-based modeling and screening approaches^61^, may enable faster optimization of EMM compositions targeting multiple drivers of pathogenesis.

Our study's key strength was using single and multicellular primary human cell systems, representing human biology and metabolism with greater fidelity than the more common immortalized cell line or animal models^62^. Importantly, we established culture conditions for our model systems to match the amino acid levels found in healthy human plasma (Supplementary Table S1), thus providing physiologically relevant condition(s) to assess the effects of LIVRQNac and its constituent amino acids. This is particularly critical when studying EMMs since the traditional nutrient-rich media^34, 63^ can mask their metabolic regulation and unique signaling properties. While primary cell systems can be influenced by donors' characteristics and limit the representativeness of findings, we helped mitigate this by routinely using cells from multiple human donors. A limitation of our study is that we are looking at complex interdependent metabolic states in an in vitro system, which may have an impact on the outcome of certain agents like the BCAAs which have systemic effects and are primarily metabolized in the muscle, and thus, may have different activity in these systems compared to in vivo. Results from our clinical studies using LIVRQNac (AXA1125) have confirmed our hypothesis, establishing the coordinated and multitargeted biological activity of this novel EMM composition to modulate the core pathogenesis of NAFLD/NASH.

In summary, this study provides evidence that LIVRQNac simultaneously and directly targets metabolic, inflammatory, and fibrotic pathways associated with NAFLD/NASH. The data also bolsters the hypothesis that the combination of specific amino acids (LIVRQNac) can drive multifactorial effects greater than those achievable by individual constituent amino acids. Collectively, this highlights the therapeutic potential of specific and potent multitargeted amino acid combinations and supports further exploration of AXA1125 in additional clinical trials for the treatment of NASH.

## Materials and methods

### PHH lipotoxicity model

Qualified PHHs from four healthy human donors (Lonza, Sekisui Xenotech, LLC.) were thawed and plated in collagen-coated plates (*Note: no human donors were directly involved in the study and PHH cells were isolated by the vendors from organ donors*). After undergoing culture and treatment for the first 2 days, the prepared PHHs were switched to an amino acid-free WEM containing defined custom amino acid concentrations that matched those found in healthy human plasma (values published in the HMDB^35^; 1× HMDB WEM; Supplementary Table S1) on Day 3 and were pretreated for 24 h with either LIVRQNac at a range of concentrations (10×–30× for LIVRQ; 2.5–7.5 mM Nac; Supplementary Table S1) or phosphate buffer saline (PBS [vehicle]). Following the pretreatment, cells were switched to media containing a lipotoxic insult (FFA 1x HMDB WEM), consisting of 0.25 mM sFFA (2:1 oleate: palmitate) + 1 ng/mL TNF-α^31^, or to the corresponding media lacking lipotoxic insult (vehicle 1× HMDB WEM) as appropriate treatments were refreshed. Twenty-four hours after lipotoxic insult, PHH supernatants were collected for chemokine analysis (MCP-1, to measure the impact of LIVRQNac on inflammatory responses of PHHs), and appropriate media and treatments were reapplied for an additional 48 h for a total of 72 h. The supernatant was collected for ApoB, urea, and ALT measurement, and cells were fixed for nuclei and lipid staining or harvested for triglyceride measurements. Detailed methods for PHH cell selection, culture and treatment, nuclei, lipid staining and microscopy, measurement of intracellular triglyceride, and PHH secreted analytes (ApoB, urea, ALT and MCP-1) are described in the Supplementary Methods Sect. 1.1.

### PHM inflammation model

Differentiated M1 and M2 macrophages were thawed and plated in a complete macrophage medium consisting of DMEM (Gibco) and allowed to recover for 24 h. Following recovery, cells were switched to amino acid-free DMEM (US Biologicals) containing custom amino acid concentrations that matched those found in healthy human plasma (values published in the HMDB^35^; 1x HMDB DMEM; Supplementary Table S1) and were pretreated with either PBS (vehicle) or LIVRQNac at a range of concentrations (10×–30× relative to human plasma levels for LIVRQ, with Nac 2.5–7.5 mM; Supplementary Table S1). Following 24 h pretreatment, M1 PHMs were stimulated with LPS (0.15 ng/mL; Sigma-Aldrich), whereas M2 PHMs were stimulated with IL-4 (1 ng/mL; Peprotech) for 24 h in the presence of corresponding concentrations of LIVRQNac or PBS. Supernatants were then collected from both M1 and M2 cells, and cytokine levels were quantified using enzyme-linked immunosorbent assay (ELISA). Detailed methods for PHM cell preparation, culture and treatment, and measurement of PHM secreted cytokines (IL-6 and TNF-α from M1 and CCL17 and CCL18 from M2 PHM) and immunofluorescence staining with HLA-DR for M1 PHMs and CD163 and CD206 for M2 PHMs following LPS (0.15 ng/mL) or IL-4 (1 ng/mL) stimulation, respectively for 24 h to assess adequate polarization phenotype are described in the Supplementary Methods Sect. 1.2.

### Primary human HSC fibrosis model

Primary HSCs were seeded into collagen I-coated plates and allowed to recover for 24 h before being switched to an amino acid-free DMEM (US Biologicals) containing a defined custom amino acid concentration that matched those found in healthy human plasma (values published in the HMDB^35^; 1× HMDB DMEM; Supplementary Table S1). HSCs were then pretreated for 24 h with either LIVRQNac at a range of concentrations (10×–20× relative to human plasma levels for LIVRQ and 2.5–5 mM Nac) or PBS (vehicle) followed by a 24-h stimulation with TGF-β1 (3 ng/mL [Peprotech]) to induce fibrosis in the presence of corresponding concentrations of LIVRQNac or PBS. TGF-β1-induced fibrogenesis was assessed by quantitative immunostaining of α-SMA, by an enzymatic measurement ELISA for procollagens 1 and 3, by quantitative reverse transcription-polymerase chain reaction of HSP47, or by EdU quantitative staining for cellular proliferation. Detailed HSC culturing and treatment methods, measurement of α-SMA, EdU positive/nuclei, HSC secreted procollagen 1 and 3, and HSP47 gene expression is described in the Supplementary Methods Sect. 1.3.

### Liver multicellular cell model

PHHs were cocultured with M1 PHMs and HSCs separated by a synthetic porous membrane in transwell plates (Fig. 4a). Following a 24-h pretreatment of the cocultured cells with LIVRQNac (30× relative to human plasma levels for LIVRQ and 7.5 mM Nac) or with the corresponding PBS (vehicle), cells were exposed to a lipotoxic insult (0.25 mM sFFA [2:1 oleate: palmitate] + TNF-α [1 ng/mL]) or the corresponding media lacking lipotoxic insult (vehicle) as appropriate treatments were refreshed. The ability of LIVRQNac to modulate secreted levels of inflammatory cytokines/chemokines (IL-6, MCP-1) and fibrosis (procollagen 1) markers was assessed by multiplex ELISA (methods described in detail in the Supplementary Methods Sect. 1.4).

### META-rank approach to determine the effectiveness of the complete composition of LIVRQNac versus individual amino acids

To deconvolute the relative contribution of the constituent amino acids of LIVRQNac to the overall activity of the complete composition, the effects of LIVRQNac and constituent amino acids (L, I, V, R, Q, Nac) or a combination (LIV) were compared on several disease-relevant phenotypes across PHH, PHM, and HSC cell models using an unbiased desirability-based, multi-objective optimization, global ranking approach (META-rank). Cells were cultured in their corresponding media containing a defined custom amino acid concentration that matched those found in healthy human plasma (values published in the HMDB^35^; 1× HMDB DMEM and 1× WEM HMDB; Supplementary Table S1) and stimulated with their corresponding stimuli in the presence of LIVRQNac, its individual constituents L, I, V, R, Q, Nac, or LIV at 20× concentration relative to the normal plasma levels for LIVRQ, L, I, V, R and Q and 5 mM for Nac. In lipotoxically-stimulated PHHs, levels of ApoB, MCP-1, and urea were measured as markers of lipid transport/metabolism, cell migration/inflammation, and hepatic health/function, respectively. In LPS-stimulated M1 PHMs, IL-6 and TNF-α inflammation markers were measured, while CCL17 and CCL18 levels were measured in IL-4-stimulated M2 PHMs. Finally, in TGF-β1-stimulated HSCs, levels of procollagen 1, procollagen 3, and α-SMA were measured as key makers of fibrosis, and EdU+/nuclei as a marker of cell proliferation. For each specific phenotype, a rank was assigned to each treatment according to the desirability of its elicited effect, with 1 being the optimal treatment and 8 being the least desirable (Fig. 5a). A META-rank (mean of the ranks) for the phenotypes measured in each cell type (e.g., IL-6 and TNF-α for M1 PHMs) is then calculated, followed by a composite all-META-rank (average META-rank for each treatment across all the cell types), capturing the optimal amino acid treatment tested across all phenotypes and all cellular systems.

### Data and statistical analysis

Results were expressed as mean percent changes ± standard error of the mean for each group relative to the median of the insult-treated group. A linear mixed-effects model was fitted to raw data from multiple donors and multiple replicates using the R package linear mixed-effects models plus S4 (lme4)^64^ to compare the test article or control article-treated groups to the vehicle control group. Results were considered significant when *p* < 0.05.

### Ethics approval

No human or animal studies were performed in this study and thus no approval and/or informed consent was required.

## Supplementary Information

Supplementary Information 1.

## Data Availability

The datasets generated during and/or analyzed during the current study are available from the corresponding author on reasonable request.
