# Supplementary material for: A novel, multitargeted endogenous metabolic modulator composition impacts metabolism, inflammation, and fibrosis in nonalcoholic steatohepatitis-relevant primary human cell models
Source: Sci Rep. 2021 Jun 4;11:11861. doi: 10.1038/s41598-021-88913-1 (PMC8178416; doi:10.1038/s41598-021-88913-1)
Supplement: Supplementary file 1 — Supplementary Information 1. [file 41598_2021_88913_MOESM1_ESM.pdf]

A novel, multitargeted endogenous metabolic modulator composition impacts metabolism, inflammation, and fibrosis in nonalcoholic steatohepatitis-relevant primary human cell models

Nadine Daou[1], Andreu Viader[2], Murat Cokol[1], Arianna Nitzel[1], Manu V. Chakravarthy[1], Raffi Afeyan[3], Tony Tramontin[4], Svetlana Marukian[5], and Michael J. Hamill[1*]

[1]Axcella Health Inc., Cambridge, MA, USA.

[2]Jnana Therapeutics, Boston, MA, USA.

[3]Flagship Pioneering, Cambridge, MA, USA.

[4]Holmusk, New York, USA.

[5]Valo Health, Cambridge, MA, USA.

Nadine Daou: ndaou@axcellahealth.com
Andreu Viader: aviader@jnanatx.com
Murat Cokol: mcokol@axcellahealth.com
Arianna Nitzel: anitzel@axcellahealth.com
Manu V. Chakravarthy: mchakravarthy@axcellahealth.com
Raffi Afeyan: raffiafeyan@gmail.com
Tony Tramontin: tony.tramontin@holmusk.com
Svetlana Marukian: smarukian@gmail.com
Michael Hamill: mhamill@axcellahealth.com

***Corresponding Author**
Michael J. Hamill
Axcella Health Inc.
840 Memorial Drive
Cambridge, MA 02139
E-mail: mhamill@axcellahealth.com
Tel: 857-939-9578

Keywords: Amino acids, endogenous metabolic modulator, fibrosis, inflammation, lipotoxicity, nonalcoholic steatohepatitis.

SUPPLEMENTARY INFORMATION

Methods

1.1 Primary human hepatocyte (PHH) lipotoxicity model

Cell selection

PHHs from four healthy human donors (Supplementary Table S3) were screened and tested to select donors that met the following quality control criteria: 1) post-thaw viability >80%, 2) polarized morphology, and 3) stable albumin secretion rate for 8–10 days.

PHH culture and treatment

Qualified PHHs were thawed and plated in PHH plating media (William's E medium, WEM [Gibco] supplemented with 10% heat-inactivated fetal bovine serum [HI-FBS] [Atlanta Biologicals], 2 mM GlutaMAX [Gibco], and 0.2% primocin [Invivogen]) on Day 1 at a density of either 60,000 or 350,000 cells/well in collagen-coated, 96-well optical microplates (Thermofisher Scientific) or 24-well (Corning), collagen I-coated plates, respectively. Throughout the study, cells were incubated at 37°C with 5% carbon dioxide. Following plating, PHHs were allowed to attach for 6 hours before washing them twice and incubating overnight in PHH plating media. On Day 2, PHHs were washed twice and incubated for 24 hours in complete hepatocyte-defined medium (cHDM, hepatocyte-defined medium [Corning] supplemented with 10 ng/mL epidermal growth factor (Corning), 2 mM GlutaMAX (Gibco), and penicillin/streptomycin (100 U/mL penicillin; 100 µg/mL streptomycin [Gibco]). On Day 3, cells were washed twice with Dulbecco's phosphate-buffered saline 1X (DPBS 1x) (Gibco) then switched to an amino acid-free WEM containing defined custom amino acid concentrations that matched those found in healthy human plasma (values published in the Human Metabolome

Database [HMDB][33]; 1x HMDB WEM; Supplementary Table S1). This custom media was supplemented as needed with either LIVRQNac added at specified fold concentrations above the plasma level (10x–30x for LIVRQ; Nac is not endogenous in plasma and was proportionally scaled between 2.5–7.5 mM; Supplementary Table S1) or phosphate buffer saline (PBS [vehicle]). The increasing fold concentrations of amino acids in these studies were used to model relevant amino acid exposures while maintaining overall cell health. On Day 4, following 24 hours of pretreatment with LIVRQNac or PBS for the vehicle, cells were switched to media containing a lipotoxic insult referred to as free fatty acid ([FFA] 1x HMDB WEM), consisting of 0.25 mM saturated FFA (2:1 oleate: palmitate) + 1 ng/mL tumor necrosis factor-alpha (TNF-α), or to the corresponding media lacking FFA (vehicle 1x HMDB WEM) as appropriate treatments were refreshed.

On Day 5, following 24 hours of lipotoxic insult, PHH supernatants were collected for chemokine analysis (monocyte chemoattractant protein-1 [MCP-1], to measure the impact of LIVRQNac on inflammatory responses of PHHs), and appropriate media and treatments were reapplied for an additional 48 hours for a total of 72 hours. The supernatant was collected for apolipoprotein B (ApoB), and urea measurement and cells were fixed for lipid staining or harvested for triglyceride measurements.

Lipid stain, nuclei stain and microscopy

For lipid stain, PHHs were seeded in 96-well, collagen I-coated plates at a density of 60,000 cells and treated/stimulated as described above. On Day 7, following 72 hours of treatment + FFA insult, cells were washed twice with PBS 1x (Gibco) and fixed with 4% paraformaldehyde (Thermofisher Scientific), then stained for lipid using HCS LipidTOX Red Neutral (Thermofisher Scientific) at 1:1000 dilutions and for nuclei using Hoechst 3342 (Life

Technologies) at 4 µg/mL. LipidTOX neutral lipid stain has an extremely high affinity for neutral lipid droplets detected by fluorescence microscopy using a high-content confocal imager (ImageXpress micro confocal IXM-5160056). Cell viability was assessed by nuclear counts and images were analyzed using MetaXpress version 6.2.3.733 software.

Intracellular triglyceride measurements

For intracellular triglyceride measurement, PHHs were seeded in 24-well, collagen I-coated plates at a density of 350,000 cells and treated/stimulated as described above. On Day 7, following 72 hours of treatment plus lipotoxic insult, cells were washed once with cold PBS 1x (Gibco), scraped off, and collected into 75 µL of standard diluent (Cayman Chemical Company). Collected cells were vortexed for 1 minute at 2,500 revolutions per minute (rpm) followed by sonication (Elmasonic sonicator) two times for 1 minute each time. Supernatants were collected for triglyceride analysis after centrifugation at 1,000 rpm for 10 minutes at 4°C. Intracellular triglyceride levels were measured using an enzymatic triglyceride colorimetric assay kit (Cayman Chemical Company) following the manufacturer's recommendations. Data were normalized to the total amount of protein as determined by a bicinchoninic acid assay (Sigma Aldrich).

Measurement of PHH-secreted analytes

PHH supernatants were collected on Day 5, following the first 24 hours of insult and treatment, and assessed for MCP-1 levels or at Day 7 following 72 hours of insult and treatment and assessed for ApoB, urea and alanine aminotransferase (ALT) levels. Human chemokine ligand (MCP-1) and ApoB were measured by enzyme-linked immunosorbent assay (ELISA) (Human CCL2/MCP-1 DuoSet ELISA, [R&D Systems]; Human ApoB ELISA development kit [HRP], [Bethyl Laboratories] respectively) at 1:20 dilution in 1x reagent diluent (Reagent Ancillary

Kit2, [R&D Systems] and at 1:5 dilution in incubation buffer (1x PBS [Gibco] with 0.05% Tween [Fisher] and 0.1% BSA [US Biologicals]) respectively according to manufacturer instructions.

Urea was measured by colorimetric assay using urea nitrogen direct assay (Stan Bio). Cell supernatant was added to the blood urea nitrogen (BUN) color reagent and BUN acid reagent at 1 (color): 2 (acid) ratio and incubated for 12 min at 100°C, then for 5 min at 4°C. Absorbance was read at 520 nm according to manufacturer instructions.

Alanine aminotransferase (ALT) was measured using the human ALT Elisa Kit (Abcam) at 1:2 dilution in the sample diluent NS (Abcam) according to manufacturer instruction.

1.2 Primary human macrophage (PHM) inflammation model

M1 and M2 PHMs cell preparation

PHMs were isolated from human blood obtained from healthy donors (Supplementary Table S3). First, peripheral blood mononuclear cells were isolated from unpurified buffy coats (Research Blood Components), and a cluster of differentiation 14-positive (CD14+) cells were selected by density gradient centrifugation using an EasySep Human CD14+ Selection Kit II (STEMCELL Technologies), according to the manufacturer's protocol. Following isolation, CD14+ monocytes were differentiated into M1 PHMs or M2 PHMs by stimulation with human granulocyte-macrophage colony-stimulating factor (GM-CSF; 500 U/mL [Peprotech]) or macrophage colony-stimulating factor (M-CSF; 100 U/mL [Peprotech]), respectively, for 10–12 days. Macrophage phenotype was confirmed by expression of macrophage-specific markers, human leukocyte antigen-DR isotype, and inducible nitric oxide synthase for M1 macrophages and

CD163 and CD206 (mannose, receptor) for M2. After GM-CSF or M-CSF stimulation, cells were cryopreserved at −80°C in liquid nitrogen for further experimentation.

PHM culture and treatment

On Day 1 of the experiment, differentiated M1 and M2 macrophages were thawed and plated at 30,000 cells per well in 96-well microplates (Thermo Fisher Scientific) in a complete macrophage medium consisting of Dulbecco's modified Eagle medium (DMEM [Gibco]) supplemented with penicillin/streptomycin (100 U/mL penicillin, 100 µg/mL streptomycin [GE Healthcare]), and 10% HI-FBS (Atlanta Bio). The cells were then incubated for 6–24 hours at 37°C and 5% carbon dioxide to recover from cryopreservation. Following recovery, cells were washed once with DPBS 1x (Gibco) and treated with amino acid-free DMEM (US Biologicals) containing custom amino acid concentrations that match those found in healthy human plasma (values published in the Human Metabolome Database[43]; 1x HMDB DMEM; Supplementary Table S1). This custom medium was supplemented with 6 mM glucose (Gibco), 1 mM sodium pyruvate (Gibco), 10 mM HEPES (H4-[2-hydroxyethyl]-1-piperazineethanesulfonic acid [Gibco]), 0.2% primocin or penicillin/streptomycin (100 U/mL penicillin, 100 µg/mL streptomycin [GE Healthcare]), and either 3% dialyzed HI-FBS for M1 PHMs or 1% dialyzed HI-FBS for M2 PHMs. Besides, the medium was supplemented as needed with either LIVRQNac added at specified fold concentrations above plasma level (10x–30x for LIVRQ; 2.5–7.5 mM Nac; Supplementary Table S1) or PBS (vehicle). On Day 3, 0.15 ng/mL of LPS (Sigma-Aldrich) or 1 ng/ml of interleukin-4 (IL-4; Peprotech) were added to the pretreatment described above for M1 or M2 PHMs, respectively. On Day 4, supernatants were collected from both M1 and M2 cells for ELISA quantification of cytokines.

Measurement of PHM-secreted cytokines

M1 PHM supernatants were collected 24 hours after the addition of LPS (0.15 ng/mL) in the presence of LIVRQNac (10x–30x for LIVRQ and 2.5–7.5 mM Nac) or PBS (vehicle). Similarly, M2 PHM supernatants were also collected 24 hours after the addition of IL-4 (1 ng/mL) in the presence of LIVRQNac (10x–30x for LIVRQ and 2.5–7.5 mM Nac) or PBS (vehicle). Interleukin-6 (IL-6) and TNF-α were quantified from M1 PHM supernatant by using commercially available ELISA kits (R&D Systems) at 1:2 and 1:4 dilutions, respectively. CCL17 and CCL18 were quantified from M2 PHM supernatant by using commercially available ELISA kits (R&D Systems) at 1:5 and 1:15 dilutions, respectively.

Immunofluorescence staining

CD14+ monocytes isolated and treated with GM-CSF or M-CSF to produce M1 or M2 polarized PHMs respectively, were cultured in DMEM 1x HMDB (Supplementary Table S1) and stimulated with LPS (0.15 ng/mL) or IL-4 (1ng/ml) respectively for 24 hours to assess adequate polarization phenotype. Polarized cells were stained for nuclei using Hoechst 3342 at 4 μg/mL, M1 PHMs were stained for HLA-DR and M2 PHMs were stained for CD163 and CD206 to confirm their phenotypes. M1 PHMs were first incubated in rabbit HLA-DR antibody (3.1 μg/mL) followed by AlexaFluor 488 goat anti-rabbit immunoglobulin G (IgG) (1 μg/mL). M2 PHMs were incubated in rabbit anti-mannose receptor (CD206) antibody (1 μg/mL) and mouse anti-CD163 antibody (10 μg/mL) followed by AlexaFluor 568 goat anti rabbit IgG (heavy plus light chains [H+L]) secondary antibody (4 μg/mL) and AlexaFluor 488 goat anti-mouse IgG (H+L) secondary antibody (1 μg/mL). M1 and M2 markers were detected by fluorescence microscopy using a high-content confocal imager (ImageXpress micro confocal IXM-5160056). Images were analyzed using MetaXpress version 6.2.3.733 software.

1.3 Primary human hepatic stellate cell (HSC) fibrosis model

HSC preparation

HSCs isolated from three healthy donors (Supplementary Table S3) were grown and maintained in complete HSC medium (DMEM [Gibco] with 10% HI-FBS [Atlanta Biologicals] and 1% antibiotic-antimycotic [Gibco]) to approximately 80% confluence in T75 or T150 flasks (Corning) and used to develop the *in vitro* model at a passage below 10.

HSC culture and treatment

HSCs were seeded into collagen I-coated, 96-well optical plastic microplates (Thermofisher Scientific) and incubated overnight in DMEM (Gibco) with 2% HI-FBS (Atlanta Biologicals) and 1% antibiotic-antimycotic (Gibco). Throughout the study, cells were incubated at 37°C with 5% carbon dioxide. On Day 2, following overnight incubation, HSCs were washed twice with DPBS 1x (Gibco) and then switched to an amino acid-free DMEM (US Biologicals) containing defined custom amino acid concentrations that matched those found in healthy human plasma (values published in the HMDB[33]; 1X HMDB DMEM; Supplementary Table S1). This custom media was supplemented as needed with either LIVRQNac added at specified fold concentrations above the plasma level (10x–20x for LIVRQ; 2.5–5 mM Nac; Supplementary Table S1) or PBS (vehicle). The increasing fold concentrations of amino acids in these studies were used to model relevant amino acid exposures while maintaining overall cell health. On Day 3, following 24 hours of pretreatment, media and treatments were refreshed and supplemented with 3 ng/mL transforming growth factor-beta 1 (TGF-β1; Peprotech) to induce fibrosis. For proliferation labeling, EdU was also added at this point for all the treatments. On Day 4, after 24 hours post-TGF-β1 induction, HSC supernatants were collected for analysis of secreted fibrotic markers (procollagen 1 and 3), and cells were either fixed for staining or harvested for

ribonucleic acid (RNA) extraction to assess gene expression by quantitative reverse transcription-polymerase chain reaction (qRT-PCR).

Measurement of HSCs-secreted procollagen 1 and 3

HSCs supernatants were collected on Day 4, 24 hours after initiation of treatment with either LIVRQNac (10x–20x for LIVRQ and 2.5–5 mM Nac) or PBS (vehicle) in the presence of TGF-β1 (3 ng/mL) stimulus and were used to assess for procollagen 1 and 3 levels. Human procollagen 1 and 3 were measured by ELISA (Human Pro-Collagen I alpha 1 [DuoSet ELISA] or Human PCIII ELISA Kit [G-Biosciences], respectively) at 1:100 dilution in 1x Reagent Diluent (Reagent Ancillary Kit 2) and 1:3 dilution in sample buffer (G-Biosciences), respectively.

5-ethynyl-2'-deoxyuridine (EdU) incorporation for proliferation and α-SMA stain

On Day 4, after 24 hours of treatment with either LIVRQNac (10x–20x for LIVRQ and 2.5–5 mM Nac) or PBS (vehicle) in the presence of TGF-β1 (3 ng/mL) stimulus and EdU, cells were washed twice and fixed with 4% paraformaldehyde (Thermo Fisher Scientific). Fixed cells were permeabilized with 0.1% TritonX-100 (Thermo Fisher Scientific), then immunostained for alpha-smooth muscle actin (α-SMA) and labeled for EdU incorporation. For α-SMA measurement, primary anti-α-SMA monoclonal antibody (1A4) isotype immunoglobulin G 2a (IgG2a) (eBioscience) was used in conjunction with the corresponding secondary antibody goat anti-mouse IgG2a cross-adsorbed secondary antibody, Alexa Fluor 647 (Invitrogen). EdU was labeled using the Click-iT EdU Alexa Fluor 555 high content screening assay (Invitrogen) according to the manufacturer's instructions. Nuclei were labeled with Hoechst 33342 (Invitrogen) at 4 μg/mL, and cells were imaged using ImageXpress micro confocal high content imager (IXM-5160056). α-SMA labeled with Alexa Fluor 647 was detected in the Cy3 channel.

EdU labeled with Alexa Fluor 555 was seen in the Texas Red channel. Nuclei labeled with Hoechst 33342 were detected in the 4',6-diamidino-2-phenylindole channel. Image analysis was performed using MetaXpress version 6.2.3.733 software.

Heat shock protein 47 (HSP47) gene expression

After 24 hours of treatment with either LIVRQNac (10x–20x for LIVRQ and 2.5–5 mM Nac) or PBS (vehicle) in the presence of TGF-β1 (3 ng/mL), cells were washed with FCW buffer (FastLane Cell Multiplex NR Kit, Qiagen). The wash buffer was immediately removed, and the cell processing mix (containing genomic DNA wipeout buffer) was applied to lyse the cells for 10 minutes at room temperature. RNA lysate was then transferred to 96-well qRT-PCR plates, sealed, and genomic DNA was digested using a thermal cycler at 75°C for 5 minutes. RNA lysate was used to perform a 1-step qRT-PCR reaction. Gene expression of HSP47 and glyceraldehyde 3-phosphate dehydrogenase (GAPDH) was multiplexed using the HEX-fluorescent dye and FAM fluorescent dye channels, respectively, with commercially available primer-probe mixes (the Human Hsp47 Primer-Probe Set, HEX; and the Human GAPDH Primer-Probe Set, FAM from Integrated DNA Technologies). Gene expression was evaluated using the ΔΔCq method.

1.4 Liver multicellular cellular model

Cytokine and procollagen secretion in a liver triculture model

A 96-well transwell plate (Corning) was used to coculture PHHs, PHMs, and HSCs isolated from healthy donors (Supplementary Table S3). Throughout the study, cells were seeded using the Lynx Pipetting System and incubated at 37°C with 5% carbon dioxide. On Day 1, HSCs were initially seeded in PHH plating media (WEM supplemented with 10% HI-FBS, 2 mM

GlutaMAX, and 0.2% primocin) at a density of 2,800 cells per transwell on the undersurface of the membrane of the transwell, which was previously coated with collagen-I at a concentration of 1.5 mg/mL on the upper surface of the transwell. Following plating, HSCs were allowed to attach for 1 hour before adding M1 PHMs on top at a density of 5,600 cells per transwell. Cocultured HSCs and M1 PHMs were then allowed to attach, and the transwell was flipped. PHHs were thawed and seeded on the collagen gel on the top surface of the transwell at a density of 28,000 cells per transwell. On Day 2, cocultured cells were switched to serum-free media and incubated for 24 hours in cHDM. On Day 3, cells were washed twice with DPBS 1x and switched to an amino acid-free WEM containing defined custom amino acid concentrations that matched those found in healthy human plasma (values published in the HMDB[33]; 1x HMDB WEM; Supplementary Table S1). This custom media was supplemented as needed with LIVRQNac added at a specified-fold concentration above plasma level (30x for LIVRQ and 7.5 mM Nac) or PBS (vehicle). Increasing the fold concentrations of amino acids in these studies was used to model relevant amino acid exposures while maintaining overall cell health. On Day 4, following 24 hours of pretreatment with LIVRQNac or PBS 1x (vehicle), cells were switched to media containing a lipotoxic insult (0.25 mM sFFA [2:1 oleate: palmitate] + 1 ng/mL TNF-α; FFA 1x HMDB WEM), or to the corresponding media lacking lipotoxic insult (vehicle 1x HMDB WEM) as appropriate treatments were refreshed.

On Day 5, after 24 hours of treatment with either vehicle (vehicle 1x HMDB WEM) or lipotoxic insult (0.25 mM sFFA [2:1 oleate: palmitate] + 1 ng/mL TNF-α; FFA 1x HMDB WEM) in the presence or absence of additional LIVRQNac (30x for LIVRQ and 7.5 mM Nac), the supernatants were collected from each side of the plate (well and transwell; Fig. 4a) and analyzed

using a custom panel Fireplex Assay (Abcam) including a multiplex panel of fibroinflammatory analytes according to the manufacturer's instructions.

SUPPLEMENTARY FIGURES

Supplementary Figure S1a: Amino acid composition of human plasma retains cell ability to respond to NASH relevant stimuli in different *in vitro* cell models

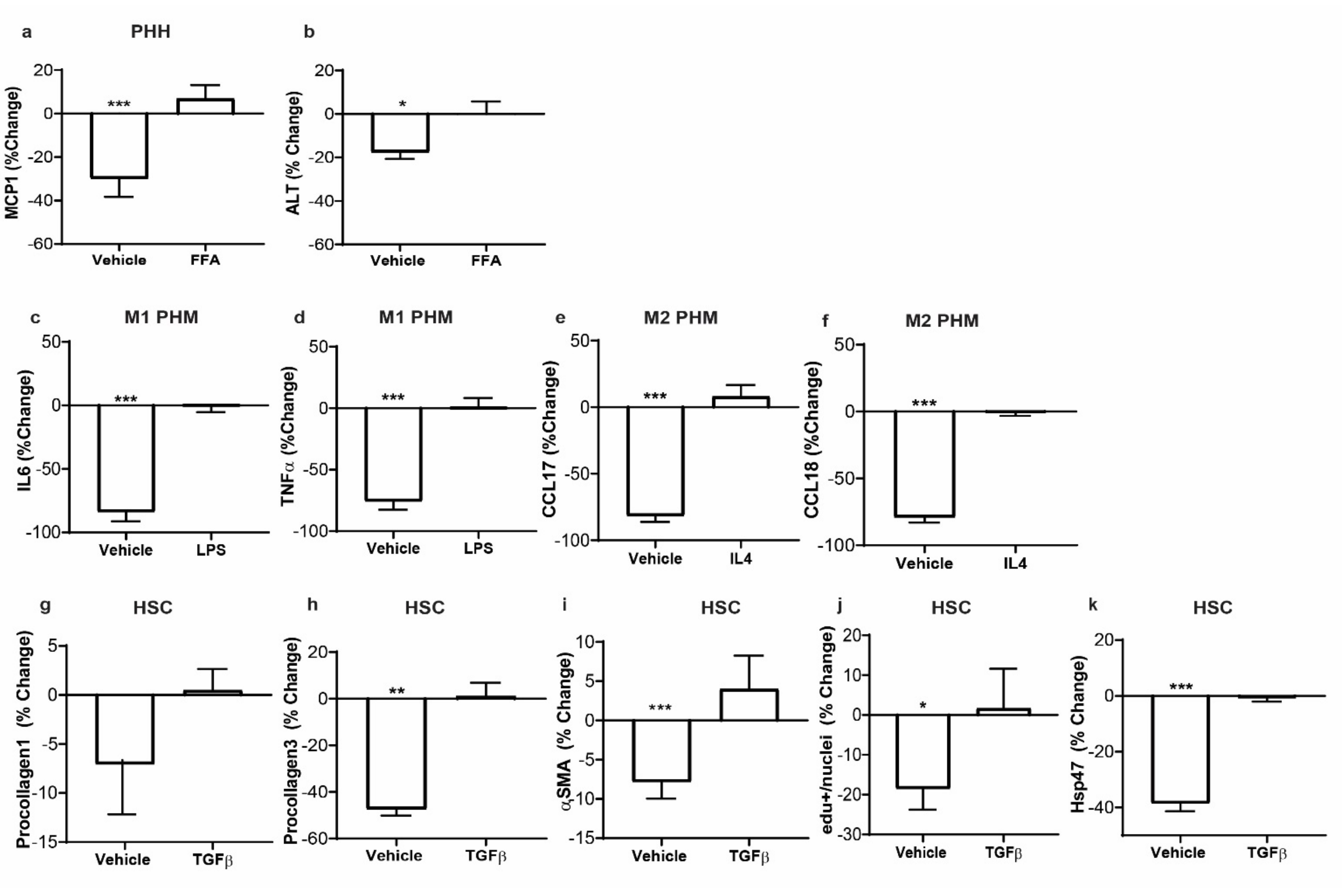

(a) MCP-1 and (b) ALT secreted levels measured in supernatants from unstimulated PHHs (vehicle) or from PHHs exposed to lipotoxic insult (FFA). Data are displayed as percent change relative to the FFA group and represent the mean of at least three technical replicates from three donors. Error bars represent ± SEM. ***p <0.001, *p <0.05 versus FFA group.

(c) IL-6 and (d) TNF-α levels were measured in supernatants from unstimulated (vehicle) or LPS (0.15 ng/mL)-stimulated M1 PHMs (LPS). Data expressed in percentage relative to LPS-stimulated M1 represent the mean of at least three technical replicates from at least five independent donors. Error bars represent ± SEM. ***p <0.001 versus LPS.

(e) CCL17 and (f) CCL18 levels were measured in supernatants from unstimulated (vehicle) or IL-4-stimulated (1 ng/mL) M2 PHMs. Data expressed in percentage relative to IL-4-stimulated M2 PHMs represent the mean of at least three technical replicates from two independent donors. Error bars represent ± SEM. ***p <0.001 versus IL-4.

(g) procollagen 1, (h) procollagen 3, (i) α-SMA, (j) EdU+/nuclei and (k) HSP-47 levels were measured in supernatants from unstimulated (vehicle) or TGF-β (3 ng/mL)-stimulated HSC (TGF-β). Data expressed in percentage relative to TGF-β-stimulated HSC represents the mean of at least three technical replicates from three independent donors. Error bars represent ± SEM. ***p <0.001, **p <0.01, *p <0.05 versus TGF-β.

Analysis was performed using GraphPad Prism version 9.0.1 for Windows, GraphPad Software, San Diego, California USA, www.graphpad.com. The graphs were assembled using Adobe Illustrator CC 2019, www.adobe.com.

ALT, alanine aminotransferase; CCL, C-C motif chemokine ligand; FFA, lipotoxic insult (0.25 mM saturated free fatty acids [2:1 oleate: palmitate] + 1 ng/mL TNF-α); HSC, hepatic stellate cells; IL, interleukin; LPS, lipopolysaccharide; MCP-1, monocyte chemoattractant protein 1; PHH, primary human hepatocyte; PHM, primary human macrophage; SEM, standard error of mean; TNF-α, tumor necrosis factor-alpha; TGF-β, transforming growth factor-beta.

Figure S1b: GM-CSF and LPS stimulation of CD14+ monocytes result in M1-specific phenotype detected by IF staining and cytokine quantification

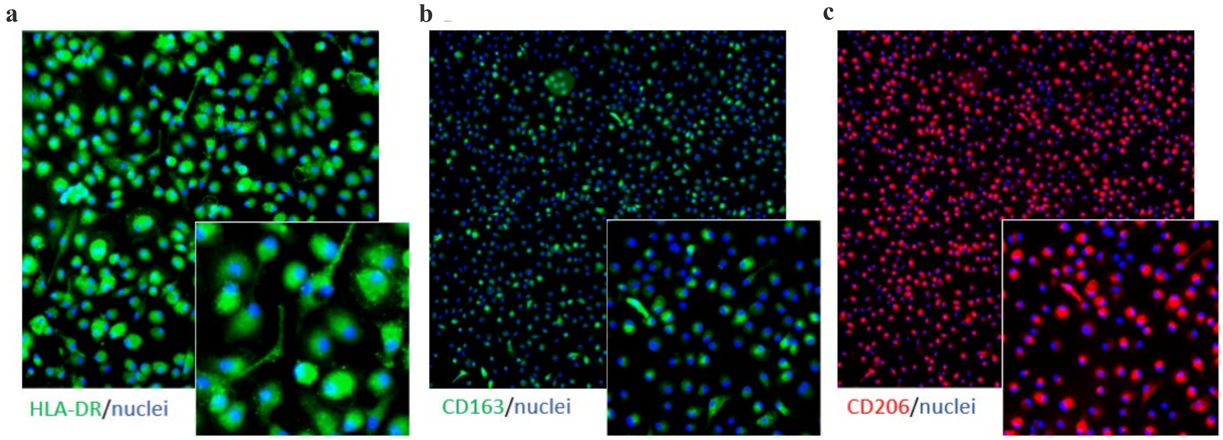

(a) Representative image of differentiated M1 PHM stimulated with 0.15 ng/mL LPS and stained for nuclei (blue), HLA-DR (green).

(b, c) Representative image of differentiated M2 PHM stimulated with 1 ng/mL IL-4 and stained for nuclei (blue, b-c), CD163 (green, b) and CD206 (red, c).

Analysis was performed using GraphPad Prism version 9.0.1 for Windows, GraphPad Software, San Diego, California USA, www.graphpad.com. The figures were assembled using Adobe Illustrator CC 2019, www.adobe.com.

CD14+, cluster of differentiation 14-positive; CD163, CD163 molecule; CD206, mannose receptor C-type 1; GM-CSF, granulocyte-macrophage colony-stimulating factor; HLA-DR, major histocompatibility complex, class II, DR alpha; IF, immunofluorescence; IL, interleukin; LPS, lipopolysaccharides; M-CSF, macrophage colony-stimulating factor; PHM, primary human macrophage.

Figure S1c: Hepatocyte cell viability assessed by nuclear counts

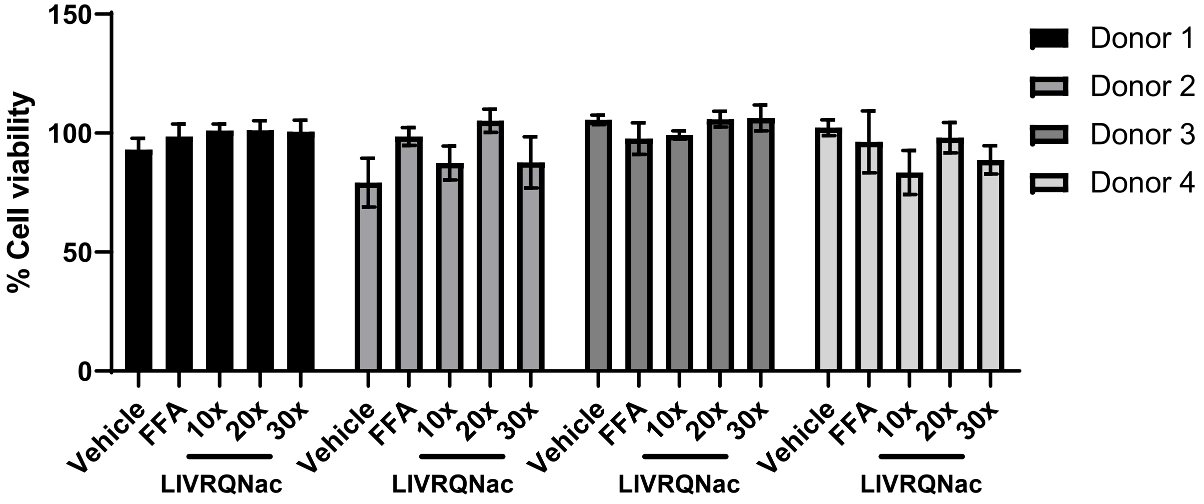

Cell viability was assessed by nuclear count of unstimulated PHHs (vehicle) or PHHs exposed to lipotoxic insult (FFA) and treated with LIVRQNac (10x–30x relative to human plasma concentration for LIVRQ and 2.5–7.5 mM Nac; applied 24 hours before lipotoxic insult) for 72 hours. Nuclear counts were analyzed by image microscopy after fixing and staining the cells with Hoechst. Data are displayed as percent change relative to the FFA treatment and represent the mean of at least three technical replicates from four independent donors. Error bars represent ± SEM. *p=0.29*.

Analysis was performed using GraphPad Prism version 9.0.1 for Windows, GraphPad Software, San Diego, California USA, www.graphpad.com. The graphs were assembled using Adobe Illustrator CC 2019, www.adobe.com.

FFA, lipotoxic insult (0.25 mM saturated free fatty acids [2:1 oleate: palmitate] + 1 ng/mL TNF-α); PHH, primary human hepatocyte; SEM, standard error of mean; TNF-α, tumor necrosis factor-alpha.

SUPPLEMENTARY TABLES

Supplementary Table S1: Amino acid concentrations based on the mean physiological concentration in the blood

	HMDB composition	LIVRQNac Composition		
**Amino Acid**	**1x (mM concentration)**	**10x (mM concentration)**	**20x (mM concentration)**	**30x (mM concentration)**
Glycine	0.251	0.251	0.251	0.251
L-Alanine	0.448	0.448	0.448	0.448
**L-Arginine**	**0.109**	**1.088**	**2.176**	**3.264**
L-Asparagine (Monohydrate)	0.048	0.048	0.048	0.048
L-Aspartic Acid	0.021	0.021	0.021	0.021
L-Cysteine (HCl Monohydrate)	0.074	0.074	0.074	0.074
L-Glutamic Acid	0.063	0.063	0.063	0.063
**L-Glutamine**	**0.562**	**5.621**	**11.242**	**16.863**
L-Histidine	0.101	0.101	0.101	0.101
**L-Isoleucine**	**0.066**	**0.6639**	**1.3278**	**1.9917**
**L-Leucine**	**0.153**	**1.527**	**3.054**	**4.581**
L-Lysine (Monohydrate)	0.219	0.219	0.219	0.219
L-Methionine	0.028	0.028	0.028	0.028
L-Phenylalanine	0.074	0.074	0.074	0.074
L-Proline	0.190	0.190	0.190	0.190
L-Serine	0.142	0.142	0.142	0.142
L-Threonine	0.151	0.151	0.151	0.151
L-Tryptophan	0.060	0.060	0.060	0.060
L-Tyrosine (Disodium Salt Hydrate)	0.151	0.151	0.151	0.151
**L-Valine**	**0.234**	**2.342**	**4.684**	**7.026**
**N-acetylcysteine**	**0**	**2.5**	**5**	**7.5**

Media baseline amino concentrations (1x). Concentrations match mean physiological levels found in human plasma (Values published in the HMDB[33]. LIVRQNac constituents (**bold**) were added at specified-fold concentrations above the plasma level (10x–30x for LIVRQ; 2.5–7.5 mM Nac). The increasing-fold concentrations of amino acids in these studies were used to model amino acid exposures while maintaining overall cell health.

HMDB, Human Metabolome Database; Nac, N-acetylcysteine.

Supplementary Table S2: Complete data (expressed as log_2 of fold change relative to FFA condition) of META-rank heat map

EMMs	Hepatocytes			M1 Macrophages		M2 Macrophages		Hepatic stellate cells			
	ApoB	MCP-1	Urea	IL-6	TNF-α	CCL17	CCL18	Procollagen1	Procollagen3	α-SMA	EdU+/nuclei
**I**	-0.038	-0.089	-0.027	-0.049	-0.063	0.268	0.326	0.151	-0.185	-0.048	-0.311
**L**	0.183	0.161	-0.223	0.059	0.149	0.218	0.533	0.251	0.057	-0.103	-0.247
**LIV**	-0.116	-0.035	-0.980	0.116	0.236	0.608	0.464	0.299	0.066	-0.114	-0.386
**LIVRQNac**	-0.393	-2.105	1.348	-0.655	-1.408	-1.675	2.043	-0.242	-0.640	-0.320	-1.443
**Nac**	0.357	-1.263	-0.180	-1.395	-2.324	-1.375	0.667	-0.245	-0.861	-0.298	-0.610
**Q**	-0.463	-0.715	0.166	0.640	0.155	0.059	0.932	0.122	-0.428	-0.035	-1.204
**R**	-0.136	0.170	1.424	-0.053	-0.022	-0.171	0.141	0.274	0.009	-0.198	-0.678
**V**	-0.175	-0.140	-0.091	-0.272	-0.083	0.257	0.240	0.155	-0.195	-0.130	-0.522

ApoB, apolipoprotein B; CCL, C-C motif chemokine ligand; EdU, 5-ethynyl-2′-deoxyuridine; EMM, endogenous metabolic modulator; IL, interleukin; MCP-1, monocyte chemoattractant protein 1; Nac, N-acetylcysteine; TNF-α, tumor necrosis factor-alpha; α-SMA, alpha-smooth muscle actin.

Supplementary Table S3: Primary cells donors sourcing and information

Donor ID	Cell Type	Vendor/Source	Sex	Race	Age	BMI
**HUM4218**	Primary Human Hepatocytes	Lonza	Male	Caucasian	19	15.1
**HUM4133**	Primary Human Hepatocytes	Lonza	Female	Caucasian	45	20.76
**HC7-12**	Primary Human Hepatocytes	Sekisui Xenotech, LLC	Male	Caucasian	61	21.4
**HC10-3**	Primary Human Hepatocytes	Sekisui Xenotech, LLC	Female	Caucasian	31	26.2
**HL160020SC**	Primary Human Hepatic Stellate Cells	Samsara Sciences	Male	Caucasian	43	23.8
**HL150002SC**	Primary Human Hepatic Stellate Cells	Samsara Sciences	Male	Caucasian	24	22.9
**HL170047SC**	Primary Human Hepatic Stellate Cells	Samsara Sciences	Male	Caucasian	34	23
**KP50962**	Human M1 Macrophage	Research Blood Components	Male	Caucasian	32	26
**KP51226**	Human M1 Macrophage	Research Blood Components	Male	Caucasian	33	26.6
**KP52838**	Human M1 Macrophage	Research Blood Components	Male	Caucasian	27	21.6
**KP52961**	Human M1 Macrophage	Research Blood Components	Male	Caucasian	20	22.3
**KP50856**	Human M1 Macrophage	Research Blood Components	Male	Caucasian	23	19.4
**KP52872**	Human M1 Macrophage	Research Blood Components	Male	Caucasian	26	25
**KP51495**	Human M1 Macrophage	Research Blood Components	Male	Caucasian	56	24.2
**KP53585**	Human M2 Macrophage	Research Blood Components	Male	Caucasian	31	25.1
**KP53443**	Human M2 Macrophage	Research Blood Components	Male	Caucasian	35	23
**KP54540**	Human M2 Macrophage	Research Blood Components	Male	Caucasian	34	22.2
**KP54788**	Human M2 Macrophage	Research Blood Components	Male	Caucasian	30	24.5

BMI, body mass index
